# Modulation of *Haemophilus influenzae* interaction with hydrophobic molecules by the VacJ/MlaA lipoprotein impacts strongly on its interplay with the airways

**DOI:** 10.1038/s41598-018-25232-y

**Published:** 2018-05-02

**Authors:** Ariadna Fernández-Calvet, Irene Rodríguez-Arce, Goizeder Almagro, Javier Moleres, Begoña Euba, Lucía Caballero, Sara Martí, José Ramos-Vivas, Toby Leigh Bartholomew, Xabier Morales, Carlos Ortíz-de-Solórzano, José Enrique Yuste, José Antonio Bengoechea, Raquel Conde-Álvarez, Junkal Garmendia

**Affiliations:** 1 Instituto de Agrobiotecnología, CSIC-Universidad Pública Navarra-Gobierno, Navarra, Spain; 2 Centro de Investigación Biomédica en Red de Enfermedades Respiratorias (CIBERES), Madrid, Spain; 3 Departamento Microbiología, Hospital Universitari Bellvitge, University of Barcelona, IDIBELL, Barcelona, Spain; 4 Servicio Microbiología, Hospital Universitario Marqués de Valdecilla and Instituto de Investigación Marqués de Valdecilla (IDIVAL), Santander, Spain; 5 Red Española de Investigación en Patología Infecciosa (REIPI), ISCIII, Madrid, Spain; 6 Wellcome-Wolfson Institute for Experimental Medicine, Queen’s University Belfast, Belfast, UK; 7 Laboratory of Preclinical Models and Analytical Tools, Division of Solid Tumors and Biomarkers, Center for Applied Medical Research, Pamplona, Spain; 8 Centro de Investigación Biomédica en Red de Enfermedades Oncológicas (CIBERONC), Madrid, Spain; 9 Centro Nacional de Microbiología, Instituto de Salud Carlos III (ISCIII), Madrid, Spain; 10 Instituto de Salud Tropical, Instituto de Investigación Sanitaria de Navarra and Dpto. Microbiología y Parasitología, Universidad de Navarra, Edificio de Investigación, Pamplona, Spain

## Abstract

Airway infection by nontypeable *Haemophilus influenzae* (NTHi) associates to chronic obstructive pulmonary disease (COPD) exacerbation and asthma neutrophilic airway inflammation. Lipids are key inflammatory mediators in these disease conditions and consequently, NTHi may encounter free fatty acids during airway persistence. However, molecular information on the interplay NTHi-free fatty acids is limited, and we lack evidence on the importance of such interaction to infection. Maintenance of the outer membrane lipid asymmetry may play an essential role in NTHi barrier function and interaction with hydrophobic molecules. VacJ/MlaA-MlaBCDEF prevents phospholipid accumulation at the bacterial surface, being the only system involved in maintaining membrane asymmetry identified in NTHi. We assessed the relationship among the NTHi VacJ/MlaA outer membrane lipoprotein, bacterial and exogenous fatty acids, and respiratory infection. The *vacJ/mlaA* gene inactivation increased NTHi fatty acid and phospholipid global content and fatty acyl specific species, which in turn increased bacterial susceptibility to hydrophobic antimicrobials, decreased NTHi epithelial infection, and increased clearance during pulmonary infection in mice with both normal lung function and emphysema, maybe related to their shared lung fatty acid profiles. Altogether, we provide evidence for VacJ/MlaA as a key bacterial factor modulating NTHi survival at the human airway upon exposure to hydrophobic molecules.

## Introduction

The Gram-negative coccobacillus nontypeable (non-capsulated) *Haemophilus influenzae* (NTHi) is a common commensal in the nasopharynx of healthy humans, but also an opportunistic pathogen causing respiratory infections such as acute otitis media, otitis media with effusion, community-acquired pneumonia, conjunctivitis and, occasionally, bacteraemia. NTHi colonization is also associated with exacerbations of chronic obstructive pulmonary disease (COPD) and with neutrophilic airway inflammation in asthma patients. Since the introduction of a vaccine against *H. influenzae* serotype b in the 1990s, the burden of *H. influenzae*-related infections has been increasingly dominated by nontypeable strains. Thus, understanding the ability of this pathogen to colonize and cause infection is an expanding area of study. Current knowledge suggests that NTHi survival in the host is a multifaceted process coordinately orchestrated by a whole range of virulence factors^1–3^.

COPD is a progressive and not fully reversible airflow limitation accompanied by emphysema, fibrosis, neutrophil infiltration and mucus hypersecretion^4^; conversely, asthma is an airway chronic inflammatory condition distinguished by abnormal immune responses leading to recurrent cough, wheezing and breathlessness^5^. Existing evidence suggests the involvement of several lipids as key molecular mediators of disease in emphysema, chronic bronchitis and asthma^6^. In fact, arachidonic acid metabolites are key players in COPD and asthma-related airway inflammation^7^, and oleic acid is associated with pulmonary injury, a trademark of acute respiratory distress syndrome^8,9^. Thus, NTHi is likely to encounter and/or interact with free fatty acids during infection of the human lower airways. Of note, a bactericidal effect of arachidonic acid has been shown for *H. influenzae*^10^; in turn, this pathogen should be equipped with mechanisms to preserve barrier function, therefore modulating its interaction with free fatty acids.

*H. influenzae* contains gene homologs to the *Escherichia coli fadL* and *fadD* genes, encoding an exogenous long-chain fatty transporter and an acyl-CoA synthase/fatty acid-CoA ligase, respectively, but it lacks those encoding the enzymes involved in fatty acid degradation via β-oxidation^11^. As a consequence, NTHi is unlikely to use free fatty acids as sole carbon and energy source, but just to synthesize phospholipids. Likewise, *H. influenzae* contains gene homologs to the *fabABDGHIZ* and *accABCD* fatty acid biosynthetic genes, and to the *plsB* and *plsC* genes, encoding two acyltransferases involved in generation of phosphatidic acid, the precursor to all phospholipids, shown to be phosphatidyl ethanolamine (PE) and phosphatidyl glycerol (PG) in *H. influenzae*^11,12^.

Phospholipids are part of the inner membrane and the inner leaflet of the outer membrane of Gram negative bacteria. The maintenance of the outer membrane lipid asymmetry is critical for its permeability barrier function^13,14^. To prevent damage resulting from surface-exposed phospholipids, *E. coli* has the phospholipase PldA, which removes the *sn*-1 and *sn*-2 fatty acid side chains from phospholipids and lysophospholipids (EC 3.1.1.32), the palmitoyltransferase PagP, cleaving palmitate of a suitable phospholipid to transfer it to lipid A (EC 2.3.1.251), and the Mla ABC transport system (maintenance of the lipid asymmetry, *vacJ*/*mlaA*-*mlaBCDEF* system), facilitating a retrograde phospholipid transport from the outer- back to the inner membrane^15^. VacJ/MlaA_*E. coli*_ is a lipoprotein acting in concert with the osmoporin OmpC at the bacterial outer membrane. Moreover, Mla system deficiency is suppressed by increased levels of PldA, and compensated by PagP mediated conversion of lipid A from the hexa- to hepta-acylated form^15,16^. *H. influenzae* contains a Mla pathway homolog, but it lacks PldA and PagP homologs. VacJ/MlaA (hereafter VacJ) mediated maintenance of the outer membrane stability is important for NTHi serum resistance, by limiting the recognition of surface oligosaccharide epitopes by natural IgM, which promotes killing via the classical pathway of complement activation^17^. This observation may relate to the proposed *vacJ* gene implication in lung pathogenesis^18^.

Together, the existing evidence prompted us to hypothesize that VacJ function may also be important to modulate NTHi interplay with hydrophobic antimicrobials, by maintaining bacterial fatty acid-phospholipid content and surface properties, therefore facilitating the human airway infection. To investigate the relationship among VacJ, fatty acids and respiratory infection by NTHi, we generated *vacJ* mutant and chromosome complemented strains by employing two genome sequenced strains, NTHi strain 375, hereafter NTHi375, and *H. influenzae* (Hi) RdKW20^11,19^. We evaluated the effects of this mutation on bacterial growth, morphology, membrane integrity and hydrophobicity, fatty acid and phospholipid composition, lipooligosaccharide (LOS) lipid A structure and phosphorylcholine (ChoP) decoration, antimicrobial resistance, and on the NTHi-host interplay by using cultured airway epithelia and a murine model of respiratory infection with normal lung function or induced emphysema. In this study, we demonstrate that inactivation of the *vacJ* gene increased bacterial fatty acid and phospholipid content, lowered NTHi resistance to a whole range of hydrophobic molecules, and resulted in reduced epithelial infection and murine lung enhanced bacterial clearance. Together, this work provides compelling evidence for VacJ being a key bacterial factor playing an important role in the NTHi-host interplay at the airways.

## Results

### Generation and characterization of *vacJ* mutant strains in *H. influenzae*

**Figure 1:**
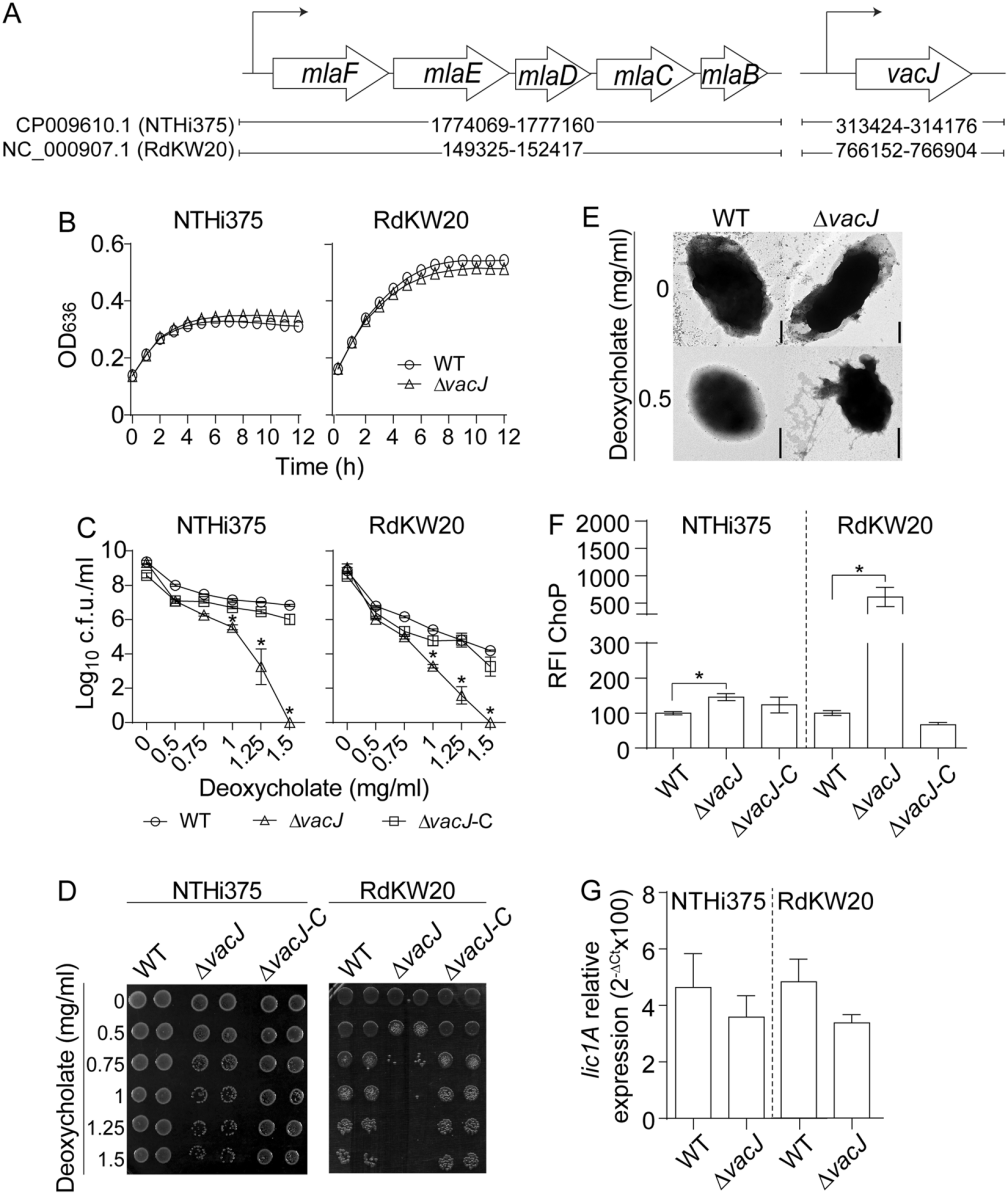
Characterization of NTHi *vacJ* mutants. (**A**) Schematic representation of *vacJ* and *mlaBCDEF* genome distribution in strains NTHi375 and RdKW20. (**B**) *H. influenzae* growth in sBHI is not modified by *vacJ* gene mutation. Bacterial growth is shown for NTHi375 (left) and RdKW20 (right) WT and *vacJ* mutant strains. Growth in sBHI is shown as a mean of OD_636_ at the indicated time points. Experiments were performed in triplicate, in three independent occasions (n = 9). (**C**–**E**) Sodium deoxycholate bactericidal effect on NTHi depends on VacJ. NTHi WT and Δ*vacJ* strains grown on chocolate agar were used to generate OD_600_-normalized bacterial suspensions for further incubation with sodium deoxycholate. After 20 min, deoxycholate dose dependent bactericidal effect was higher on the Δ*vacJ* mutant than on the WT strains, as measured by assessing bacterial growth on sBHI (**C,D**). In (**C**), bacterial counts are shown as log_10_ c.f.u./ml (mean ± SE). For both NTHi375 and RdKW20, *vacJ* mutant viability was lower than WT strain at [deoxycholate] between 1 and 1.5 mg/ml (*p < 0.0005). Experiments were performed in duplicate and in three independent occasions (n = 6). Statistical comparison of the means was carried out using one-way ANOVA (Dunnett’s multiple comparisons test). (**E**) Inactivation of the *vacJ* gene altered bacterial morphology in the presence of deoxycholate, observed by TEM. NTHi375 WT (left) and Δ*vacJ* mutant (right) strains are shown. Upper panels: examples of transmission electron micrographs of negatively stained bacteria grown for 12 h on chocolate agar at 37 °C with 5% CO_2_. Original magnification ×60.000. Bars: 0.25 µm. Lower panels: examples of transmission electron micrographs of negatively stained bacteria grown as indicated above, washed in PBS and incubated with 0.5 mg/ml deoxycholate for 20 min. Original magnification ×50.000; Bars: 0.5 µm. (**F**) VacJ modulates NTHi ChoP level. WT and Δ*vacJ* strains were exponentially grown in sBHI, incubated with anti-ChoP antibody, and antibody binding was analysed by flow cytometry. ChoP detection was higher in Δ*vacJ* than in the WT strains (mean ± SE, *p < 0.005). Experiments were performed in quadruplicate and in three independent occasions (n = 12). Statistical comparison of the means with one-way ANOVA (Dunnett’s multiple comparisons test) was carried out. (**G**) Comparable expression of the *lic1A* gene between WT and *vacJ* mutant strains, exponentially grown in sBHI. Experiments were performed in duplicate and in three times (n = 6). Statistical comparison of the means using two-tail *t*-test was performed.

Two-hundred and ninety eight available nontypeable *H. influenzae* whole genome sequences were downloaded and used to assess the presence of the Mla system through the TBLASTN tool by local BLAST. Gene homologs to those encoding the *E. coli* Mla system were shown to be present in all strains. Similar to *E. coli*, the *vacJ* gene and the *mlaBCDFE* operon occupy separate genomic locations (Fig. 1A). NTHi375 and RdKW20 strains were employed to generate *vacJ* mutants (*vacJ* gene accession numbers NF38_01540 and HI0718, respectively). The *vacJ* gene is predicted to encode a lipoprotein located at the bacterial outer membrane. VacJ_NTHi375_ and VacJ_RdKW20_ present a putative signal sequence (MKTKVILTALLSAIALTGC and MKTKTILTALLSAIALTGC, respectively) in amino acids 1–19, and a lipobox sequence (Leu-Thr-Gly-Cys) in amino acids 16–19. At the protein level, VacJ_NTHi375_ and VacJ_RdKW20_ displayed 98.4% identity; VacJ_NTHi_ presents homologs in other Gram-negative bacteria^20^.

NTHi mutants lacking the *vacJ* gene were selected on sBHI agar. These strains rendered normal size colonies (Fig. S1A). Δ*vacJ* mutants growth in sBHI was comparable to that of their respective isogenic wild type (WT) strains (Fig. 1B). Similar bacterial morphology was also observed for WT and mutant strains when assessed by transmission electron microscopy (TEM) (Fig. 1E). Deletion of the *vacJ* gene is known to impair outer membrane stability in several Gram negative bacteria including *H. influenzae*, leading to increased detergent sensitivity^17,21–23^. In agreement, in the presence of sodium deoxycholate, both NTHi375 and RdKW20 *vacJ* mutants showed bacterial cell morphology changes, and a sodium deoxycholate dose dependent reduction of viability, which could be chromosomally complemented (Figs 1C–E and S1B).

Moreover, it has been previously shown that mutation of the *vacJ* gene in *H. influenzae* strain R2866 increases the amount of ChoP on the LOS molecule^17^. NTHi375Δ*vacJ* and RdKW20Δ*vacJ* strains also showed significantly higher ChoP level than their respective WT strains (p < 0.005) when measured by flow cytometry with the murine monoclonal antibody TEPC-15, and this phenotype could be restored to WT levels in the Δ*vacJ* complemented strains (Fig. 1F). We asked if such ChoP increase could be related to the *lic1A* gene phase variation or to changes in *lic1ABCD* gene expression. The *lic1A* gene was sequenced in WT and *vacJ* mutant strains, showing the same number of repeats of the tetranucleotide (5′CAAT)_n_ (Fig. S1C); similarly, *lic1A* gene expression was comparable between WT and *vacJ* mutant strains (Fig. 1G).

In summary, disruption of the *vacJ* gene did not modify NTHi morphology and growth under the conditions tested; differently, it affected NTHi outer membrane stability and increased bacterial surface decoration with ChoP, independently of the *lic1A* gene expression or phase variation.

### Disruption of the *vacJ* gene modifies NTHi fatty acid content

**Figure 2:**
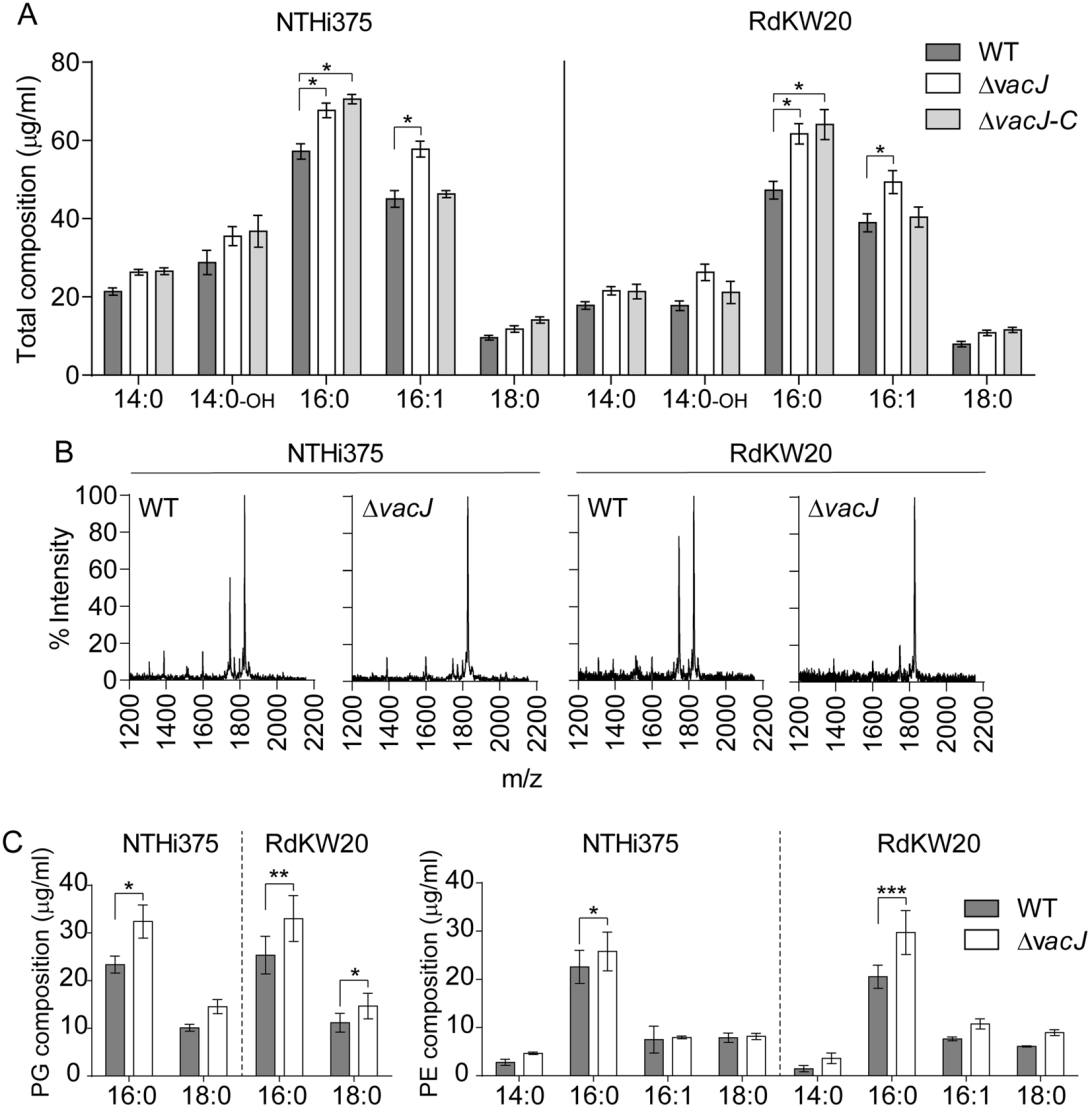
Determination of NTHi fatty acid composition by GC-MS. (**A**) Total fatty acid composition, as methyl esters (means ± SE). Bacteria grown on chocolate agar were used to extract fatty acids, by following saponification, methylation, extraction and washing steps. *vacJ* mutants showed increased amounts of C16:0 and C16:1 compared to the WT strains (for NTHi375 and RdKW20, *p < 0.0005). Δ*vacJ* complemented strains showed amounts of C16:1 comparable to those of the WT strains; increased amounts of C16:0 were maintained in the Δ*vacJ* complemented strains (for NTHi375 and RdKW20, *p < 0.0005). Quantifications were performed in duplicate and in three independent occasions (n = 6). (**B**) Determination of NTHi lipid A species by MALDI-TOF for NTHi375 (left) and RdKW20 (right) WT and *vacJ* mutant strains. (**C**) TLC band corresponding to PE and PG phospholipids were scraped to analyse their fatty acid composition. Assays were performed in duplicate and in three independent occasions (n = 6) (means ± SE are shown). PG C16:0 content was higher in the *vacJ* than in the WT strains (for NTHi375, *p < 0.05; for RdKW20, **p < 0.005). PG C18:0 content was higher in the *vacJ* than in the WT strains (for RdKW20, *p < 0.05). PE C16:0 content was also higher in the *vacJ* than in the WT strains (for NTHi375, *p < 0.05; for RdKW20, ***p < 0.0001). Statistical comparisons of the means were carried out with two-way ANOVA (Sidak’s multiple comparisons test).

The content of NTHi surface phospholipids has been shown to increase upon *vacJ* gene mutation^17^. Such modification may relate to variations in bacterial fatty acid composition, in terms of fatty acyl residues type and/or amount. Based on this notion, bacterial total fatty acid methyl ester composition was analysed by gas chromatography and mass spectrometry (GC-MS), showing increased content of total fatty acids in *vacJ* mutant compared to WT strains (for NTHi375 and RdKW20, p < 0.005) (Fig. S2A). The same type of fatty acyl residues, consisting of straight saturated C14:0 (myristic and 3-hydroxymyristic), C16:0 (palmitic), C18:0 (stearic) and unsaturated C16:1 (palmitoleic) acid was observed in all tested strains. Δ*vacJ* mutants displayed increased content of palmitic (for NTHi375 and RdKW20, p < 0.0005) and palmitoleic acid (for NTHi375 and RdKW20, p < 0.0005), compared to their WT isogenic strains. The content of palmitoleic shown by *vacJ* complemented strains was similar to that of the WT strains; this was not the case for palmitic acid content (for NTHi375 and RdKW20, p < 0.0005). No significant differences were observed for myristic, 3-hydroxymyristic (C14:0-3-OH) and stearic acid levels between WT and *vacJ* mutant strains (Fig. 2A).

The *H. influenzae* lipid A disaccharide backbone is known to be composed of two 2-amino-2-deoxyglucose residues linked by a β-(1–6) glycosidic linkage and phosphorylated at positions 1 and 4′. The C2/C2′ and C3/C3′ positions are substituted by amide-linked and ester-linked myristic acid, respectively; fatty acid chains on C2′ and C3′ are further esterified by 14:0, and fatty acid chains on C2 and C3 are 3-hydroxymyristic acid^24^. We next assessed WT and *vacJ* mutant strains lipid A species by MALDI-TOF mass spectrometry. All strains showed a major lipid A species corresponding to the hexa-acylated form (m/z 1,824)^24,25^. The ion peak corresponding to m/z 1,744, likely to represent the monophosphorylated hexa-acylated lipid A, showed lower intensity in *vacJ* mutant than in WT strains; no differences among strains were observed for the ion peak with m/z 1,388, which may represent a tetra-acyl lipid A^24,25^ (Fig. 2B).

As previously stated^12^, thin layer chromatography (TLC) analysis showed that *H. influenzae* WT and *vacJ* mutant phospholipids are phosphatidyl ethanolamine (PE) and phosphatidyl glycerol (PG) (Fig. S2B). PG showed C16:0 and C18:0 in all tested strains, and C16:0 was shown to be the most abundant one. A higher PG fatty acid content was observed, being significant for C16:0 in both Δ*vacJ* mutants (for NTHi375, p < 0.05; for RdKW20, p < 0.005), and for C18:0 in RdKW20Δ*vacJ* (p < 0.05) (Fig. 2C, left panel). PE displayed C14:0, C16:0, C16:1 and C18:0, being also C16:0 the most abundant. Δ*vacJ* mutants showed increased palmitic acid (for NTHi375, p < 0.05; for RdKW20, p < 0.0001) compared to their isogeneic WT strains (Fig. 2C, right panel).

The observed increased fatty acid content led us to assess bacterial surface hydrophobicity. Although we did not observe striking differences between isogenic WT and Δ*vacJ* strains, a slight but consistent tendency to increased bacterial surface hydrophobicity in the absence of the *vacJ* gene could be seen (Fig. S2C).

Together, our results show increased amount of total fatty acids and identify specific fatty acyl residues raised in NTHi *vacJ* mutant strains, i.e. palmitic and palmitoleic acid. Such fatty acid increase may relate to changes in bacterial phospholipid composition, without having a gross influence on lipid A acylation patterns, altogether supporting the notion that NTHi *vacJ* mutants may have increased surface hydrophobicity compared to their isogenic parental strains.

### Inactivation of the *vacJ* gene lowers NTHi resistance to hydrophobic antibiotics

The observations above prompted us to speculate that changes in bacterial surface hydrophobicity may modify antibiotic susceptibility. We tested bacterial susceptibility to 12 hydrophilic antibiotics, showing no differences between WT and mutant strains (Table S1). In contrast, NTHi375 and RdKW20 *vacJ* mutants were more susceptible to the hydrophobic antibiotics erythromycin, rifampicin and azithromycin (Table 1). In this case, phenotypic restoration could not be performed because the complemented strains are resistant to erythromycin (see Methods section). Together, bacterial changes associated to *vacJ* mutation were found to contribute increasing NTHi susceptibility to hydrophobic antibiotics.

**Table 1:** Minimal inhibitory concentrations (MIC) of three hydrophobic antibiotics and one antimicrobial peptide against NTHi WT and *vacJ* mutant strains.

Strain	Erythromycin		Rifampicin		Azithromycin		Polymyxin E
	^a^MIC (mg/l)	Diameter inhibition zone (mm)	^a^MIC (mg/l)	Diameter inhibition zone (mm)	MIC (mg/l)	Diameter inhibition zone (mm)	^a^MIC (mg/l)
NTHi375WT	8	17	0.25	25	2	19	0.094
NTHi375Δ*vacJ*	3	20	0.094	29	1	21	0.032
RdKW20WT	16	14	0.5	22	4	16	0.047–0.064
RdKW20Δ*vacJ*	4	18	0.19	23	2	19	0.032

^a^MIC (mg/l) of Erythromycin, Rifampicin and Polymyxin E obtained by E-test.

### The *vacJ* gene mutation increases *H. influenzae* susceptibility to ionic antimicrobials

Changes in resistance to hydrophobic antibiotics may also be extended to other antimicrobials with hydrophobic regions. Polymyxins consist of a cyclic heptapeptide ring with a tripeptide side chain covalently bound to a fatty acid via an acyl group. The polycationic peptide ring interacts with the lipid A, and the fatty acid portion interacts with the hydrophobic region of the outer membrane, driving loss of membrane integrity and bacterial cell death^26,27^. Following this notion, we next tested NTHi WT and mutant strains resistance to the antimicrobial peptide polymyxin E (PxE, also known as colistin). We observed increased PxE susceptibility for both Δ*vacJ* strains (MIC_PxE_ of 0.032 mg/l), compared to that of the WT strains (for NTHi375, MIC_PxE_ of 0.094 mg/l; for RdKW20, MIC_PxE_ of 0.064 mg/l) (Table 1).

### Disruption of the *vacJ* gene in *H. influenzae* increases bacterial susceptibility to free fatty acids

**Figure 3:**
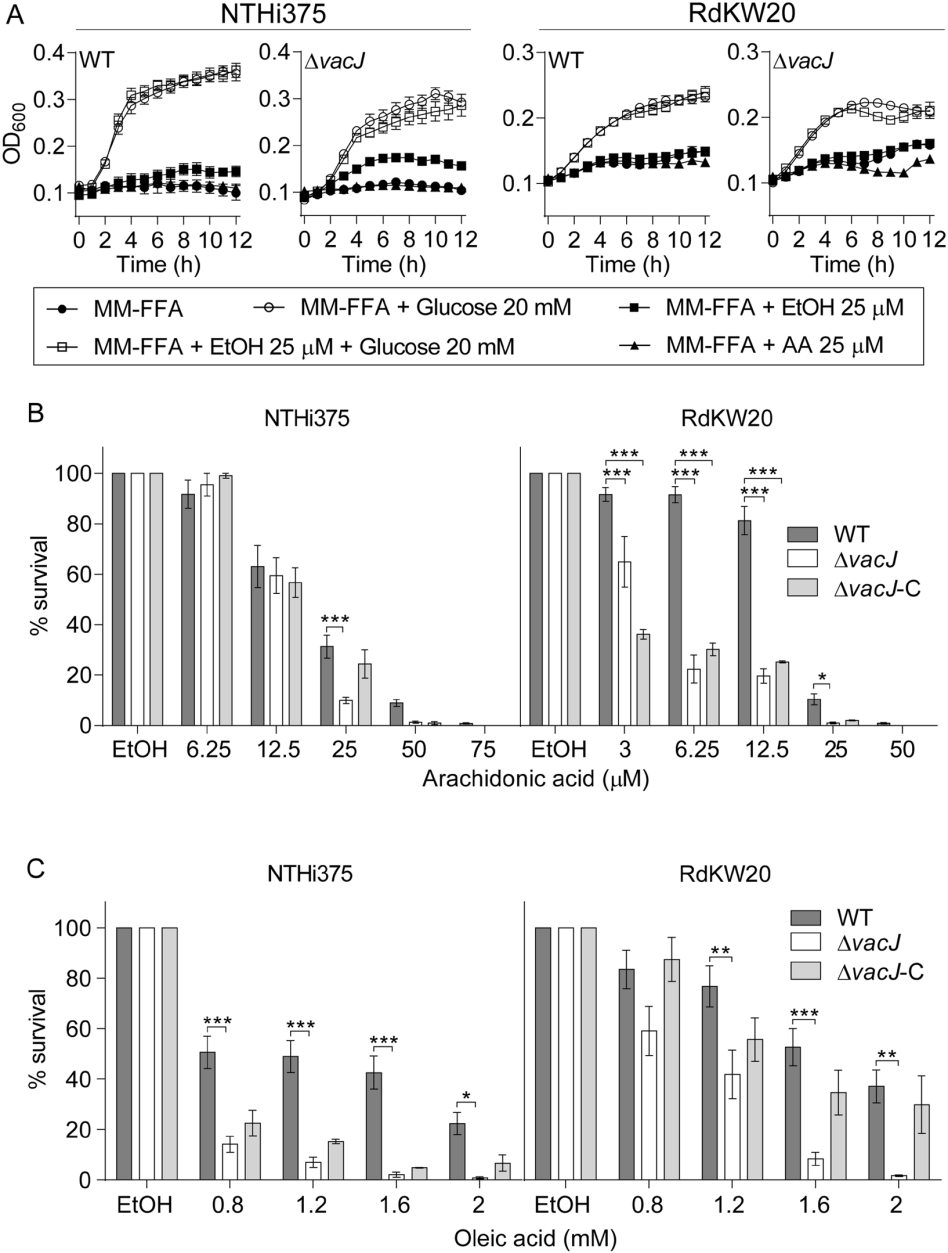
Free fatty acids are not used as carbon and energy source, and reduce NTHi viability in a VacJ dependent manner. (**A**) Growth in MM-FFA is shown as a means of OD_600_ at the indicated time points (mean ± SE). In all strains, glucose allowed exponential growth. Ethanol and arachidonic acid did not render bacterial growth, similar to only MM-FFA conditions. Growth curves were performed in quadruplicate, three times (n = 12) (**B**) Arachidonic and (**C**) oleic acid have a bactericidal effect on NTHi. WT, Δ*vacJ* and Δ*vacJ* complemented strains grown on chocolate agar were used to generate normalized bacterial suspensions in MM-FFA, for further incubation with arachidonic or oleic acid. Results are expressed as percentage of bacterial survival (means ± SE), referred to that in the presence of vehicle solution. A dose dependent bactericidal effect was observed for both WT strains and fatty acids. Both *vacJ* mutants were more susceptible to the two fatty acids tested than their respective parental strains (NTHi375, arachidonic acid 25 µM, ***p < 0.0005; NTHi375, oleic acid 0.8 to 1.6 mM, ***p < 0.0005; 2 mM, *p < 0.05; RdKW20, arachidonic acid 3 to 12.5 µM, ***p < 0.0005; 25 µM, *p < 0.05; RdKW20, oleic acid 1.2 to 2 mM, **p < 0.005). Complete or partial restoration was observed for NTHi375 and both free fatty acids, and for RdKW20 and oleic acid; increased arachidonic acid killing was maintained for the RdKW20Δ*vacJ* complemented strain (arachidonic acid 3 to 12.5 µM, ***p < 0.0005). Survival assays were performed in duplicate and at least three times (n ≥ 6). Statistical comparisons of the means were performed with two-way ANOVA (Sidak’s multiple comparisons test).

Free fatty acids are considered as natural detergents; in fact, a bactericidal effect on *H. influenzae* has been described for arachidonic acid (C20:4)^10^. The results above led us to speculate that lacking the *vacJ* gene may further increase bacterial susceptibility to fatty acids. Based on this notion, we assessed the result of NTHi interaction with exogenous fatty acids. To do so, bacterial growth in a defined medium free of fatty acids (MM-FFA), where a carbon source can be added *ad hoc*, was first optimized. Medium composition and NTHi bacterial growth when using glucose 20 mM as carbon source are shown in Table S2 and Fig. 3A, respectively. NTHi is unlikely to degrade exogenous long-chain fatty acids due to the absence of β-oxidation, therefore excluding the use of such molecules as sole carbon and energy source by this pathogen. As expected, bacterial growth in MM-FFA could not be recorded when using either arachidonic acid or vehicle solution as carbon source; *vacJ* mutants showed similar behaviour to their respective WT strains (Fig. 3A). To further determine NTHi viability in the presence of free fatty acids, NTHi WT and *vacJ* mutant strains were inoculated in MM-FFA in the presence of arachidonic (C20:4) or oleic (C18:1) acid. Such fatty acid selection was based on their presence in the human lung^7–9^. NTHi375 and RdKW20 viability decreased in the presence of both fatty acids in a dose dependent manner. Arachidonic acid susceptibility was higher for RdKW20 than for NTHi375, leading us to assess non-identical concentrations of this fatty acid for both strain backgrounds. As expected, mutant strains lacking the *vacJ* gene displayed higher susceptibility to both arachidonic and oleic acid than their respective WT strains (NTHi375, arachidonic acid 25 µM, p < 0.0005; NTHi375, oleic acid 0.8 to 1.6 mM, p < 0.0005; 2 mM, p < 0.05; RdKW20, arachidonic acid 3 to 12.5 µM, p < 0.0005; 25 µM, p < 0.05; RdKW20, oleic acid 1.2 to 2 mM, p < 0.005). (Fig. 3B,C). Complete or partial restoration was obtained for the Δ*vacJ* complemented strains, for NTHi375 and both free fatty acids, and for RdKW20 and oleic acid (Fig. 3B, left panel; Fig. 3C).

Together, these results showed that NTHi does not grow using free fatty acids as sole carbon source; in turn, free fatty acids have a killing effect, further increased by inactivation of the *vacJ* gene.

### VacJ contributes to NTHi interplay with human airway epithelial cells

**Figure 4:**
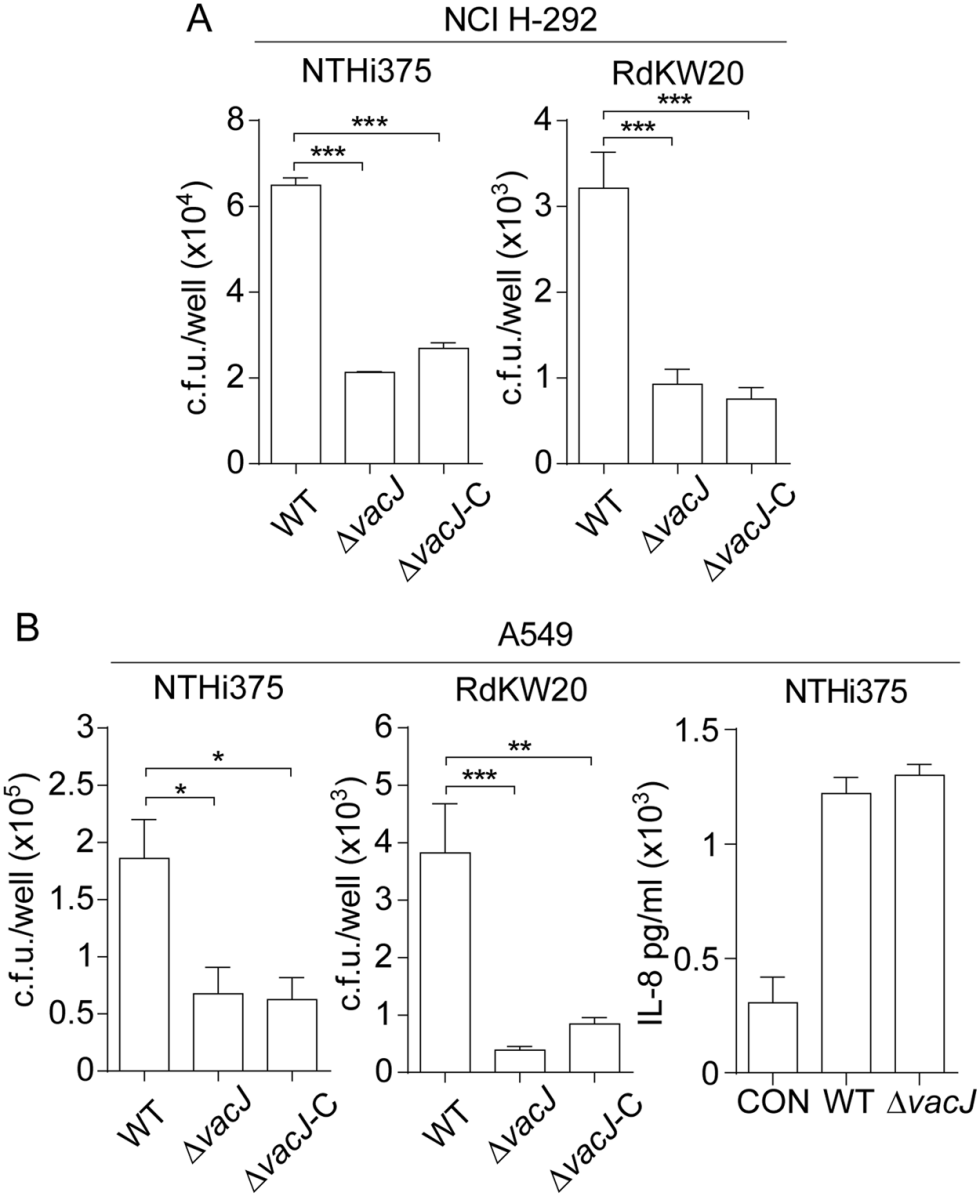
The *vacJ* gene inactivation decreases NTHi interplay with cultured human airway epithelia. (**A**) NCI-H292 bronchial epithelial cells and (**B**) A549 type II pneumocytes were used to quantify invasion by WT, Δ*vacJ* and Δ*vacJ* complemented strains. Inactivation of the *vacJ* gene triggered significantly lower entry into NCI-H292 (for NTHi375 and RdKW20, ***p < 0.0005) and A549 (for NTHi375, p < 0.05; for RdKW20, ***p < 0.0005) cells than that shown by the WT strains. Same observation was made for Δ*vacJ* complemented strains (NCI-H292 cells: for NTHi375 and RdKW20, ***p < 0.0005; A549 cells: for NTHi375, **p < 0.005; for RdKW20, ***p < 0.0005). IL-8 release was comparable in cells infected by NTHi375 WT and *vacJ* mutant strains. Cell invasion assays were carried out in triplicate, at least three times (n ≥ 9). IL-8 levels were quantified twice in two independent assays (n = 4). Means ± SD are represented and statistical comparisons of the means were performed using two-tail *t*-test.

Given the determinant role of NTHi interplay with the human airway epithelium in the progression of infection^2,28^, we speculate that VacJ mediated changes on the bacterial surface may alter such interplay. We next asked whether the lack of VacJ alters this host cell-pathogen interaction by infecting NCI-H292 human bronchial epithelial cells with NTHi WT and *vacJ* mutant strains^29^. NCI-H292 cell invasion of Δ*vacJ* mutants was lower than that shown by the WT strains (for NTHi375 and RdKW20, p < 0.0005) (Fig. 4A). Δ*vacJ* lower epithelial infection rate was also observed during invasion of A549 human type II pneumocytes (for NTHi375, p < 0.05; for RdKW20, p < 0.0005)^30,31^ (Fig. 4B, left and middle panels). Phenotypic restoration could not be achieved with the Δ*vacJ* complemented strains (Fig. 4). Differently, the *vacJ* gene mutation did not modify epithelial inflammatory response in terms of IL-8 secretion by A549 cells upon infection (Fig. 4B, right panel). In summary, VacJ is likely to contribute significantly to *H. influenzae* entry within airway epithelia.

### Inactivation of the vacJ gene attenuates *H. influenzae* virulence *in vivo*

**Figure 5:**
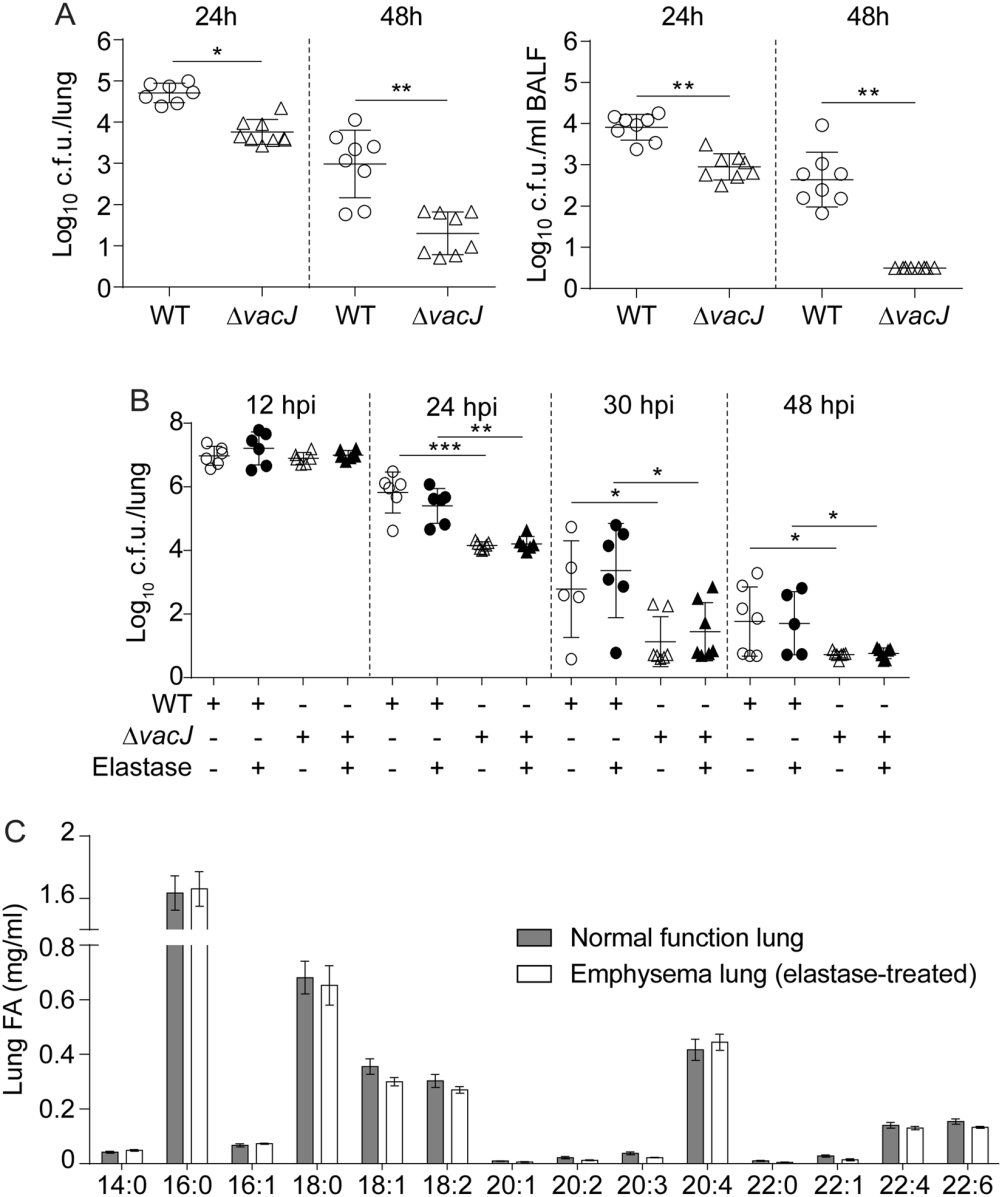
Inactivation of the *vacJ* gene attenuates *H. influenzae* virulence. (**A**) CD1 mice were intranasally infected with ~2 × 10^8^ bacteria/mouse of NTHi375 WT (circle) or Δ*vacJ* (triangle) strains. Mice were euthanized at 24 and 48 hpi, and bacterial loads quantified in lungs (left panel, log_10_ c.f.u./lung) and BALF samples (right panel, log_10_ c.f.u./ml BALF). NTHi375Δ*vacJ* showed significantly lower loads in lungs and BALFs at 24 (*p < 0.005 and **p < 0.0001, respectively) and 48 (**p < 0.0001) hpi than those shown by the WT strain. (**B**) CD1 mice were administered elastase to induce lung emphysema (vehicle solution was used as control), and intranasally infected with ~2 × 10^8^ bacteria/mouse of NTHi375 WT (circle) and Δ*vacJ* (triangle) strains. Mice were euthanized at 12, 24, 30 and 48 hpi, and bacterial loads were quantified in lungs. White and black symbols refer to bacterial loads in vehicle solution and elastase-treated animals, respectively. At 24, 30 and 48 hpi, NTHi375Δ*vacJ* showed significantly lower loads in lungs than those shown by the WT strain (in lungs with normal function, ***p < 0.0001 at 24 hpi, *p < 0.05 at 30 and 48 hpi; in emphysema lungs, **p < 0.001 at 24 hpi, *p < 0.05 at 30 and 48 hpi). (**C**) Determination of total fatty acid composition in CD1 mouse lung by GC-MS. A repertoire of fatty acid species was detected and quantified in lung samples of CD1 mice (n = 6 per group); same fatty acid profile was observed for lungs with normal function and with emphysematous lesions. Means ± SD are shown, statistical comparisons of the means were performed using two-tail *t*-test.

A genome-wide screen for *H. influenzae* genes required in the lung previously revealed *vacJ* involvement in pathogenesis^18^. Following this observation, we sought to quantify the impact of *vacJ* disruption in a NTHi mouse respiratory infection model system previously used for NTHi375^29,31–34^. Mice were infected with NTHi375 WT and *vacJ* mutant strains, and bacterial loads quantified in lung and bronchoalveolar lavage fluid (BALF) samples at 24 and 48 h post-infection (hpi). At both post-infection time points, NTHi375Δ*vacJ* lung and BALF bacterial numbers were lower than those recovered for the WT strain (at 24 hpi, lung and BALF samples, p < 0.005 and p < 0.0001 respectively; at 48 hpi, p < 0.0001) (Fig. 5A).

The use of mice with normal lung function may limit modelling NTHi infection in the context of an emphysematous lung. When tested for NTHi strain 2019, murine lung treatment with elastase resulted in damage consistent with COPD/emphysema and impaired clearance of NTHi^35^. We next speculate that *vacJ* mutant attenuation may be favoured, among others, by lung fatty acids. Lung emphysema, where fatty acids seem to mediate airway inflammation^6,7^, could in turn amplify the attenuation effect of *vacJ* disruption. To elicit pulmonary damage consistent with emphysema, CD1 mice were treated with porcine pancreatic elastase delivered via nonsurgical intratracheal instillation^36^. Emphysema-related features in elastase-treated mice were confirmed by pulmonary function tests and micro-computed tomography (micro-CT) (Fig. S3). Namely, elastase-treated lungs displayed higher physiological compliance and lower resistance and elastance than vehicle-treated mice. These results suggest an increment in the lung capacity to expand and a reduction in the resistance to the airflow, as a result of the parenchymal degradation induced by the effect of elastase activity. Moreover, micro-CT image scans revealed lower X-ray density and swollen lungs in elastase-treated mice, which again correlates with a decrease in lung stiffness and collapsed lung parenchymal structures. All these findings are consistent with those previously reported^36^. After 17 days, mice were infected with NTHi375 WT and Δ*vacJ* strains, and bacterial loads quantified in lung samples. Elastase effect on NTHi375 clearance from the lungs was first determined. While bacterial counts obtained from lung homogenates were comparable at 24 and 48 hpi, we observed a slight trend to recover higher WT bacterial numbers from elastase-treated mice at 12 and 30 hpi (Fig. 5B). As shown above, mock-treated animals rendered lower NTHi375Δ*vacJ* numbers than those recovered for the WT strain at 24 hpi (p < 0.0001), 30 (p < 0.05) and 48 (p < 0.05) hpi. The counts obtained from elastase-treated mice infected with the mutant strain were comparable to those obtained from mock-treated mice at any post-infection time point but, notably, elastase-treated animals rendered significantly lower NTHi375Δ*vacJ* numbers than those recovered for the WT strain at 24 (p < 0.001), 30 (p < 0.05) and 48 (p < 0.05) hpi (Fig. 5B). GC-MS analysis of murine lung fatty acid methyl ester composition showed the same profile of fatty acyl residues in both normal function and emphysematous lung samples, consisting of saturated myristic, palmitic, stearic and C22:0 (behenic) acids, and unsaturated palmitoleic, oleic, C18:2 (linoleic), C20:1, C20:2, C20:3, arachidonic, C22:1 (erucic), C22:4 (docosatetraenoic) and C22:6 (docosahexaenoic) acids (Fig. 5C).

Overall, these results support that VacJ is a bacterial factor involved in NTHi pulmonary infection. Under the conditions tested, elastase-treated mice showed a subtle impairment of NTHi375 pulmonary clearance; induced emphysema did not modify the dynamics of NTHi375Δ*vacJ* clearance. The panel of saturated and unsaturated fatty acids quantified in murine lung samples was comparable for normal function and emphysematous lungs, which may contribute to the observed *vacJ* mutant attenuation in both mouse infection models.

## Discussion

Lipids are important components of the host immunity by organizing membrane signalling complexes and releasing lipid-derived mediators. Changes in the lipid content of the airway epithelium play important roles in cystic fibrosis, COPD and asthma, and several lipid molecules are key molecular mediators of disease in these chronic respiratory conditions^6^. In fact, COPD is linked to dysregulation of many metabolic pathways including lipid biosynthesis, and reconstruction of a COPD sputum lipid signalling network indicates that arachidonic acid may be a critical and early signal distributer upregulated in this disease^37^. Likewise, pulmonary injury is a trademark of acute respiratory distress syndrome associated to oleic acid^8,9^. Chronic respiratory patients are often persistently infected at their lower airways by NTHi, which may encounter free fatty acids at this host niche. Free fatty acid antibacterial properties have been observed for several pathogens including group A streptococci, *Neisseria gonorrhoeae*, mycobacteria, *Staphylococcus aureus* and *H. influenzae*^10,38–42^, but mechanisms counteracting their detergent effect have not been detailed. Outer membrane lipid asymmetry plays an essential role in Gram negative bacteria barrier function. Its maintenance contributes preserving surface hydrophilicity, further counteracting the antibacterial effect of hydrophobic antimicrobials such as fatty acids and numerous conventional antibiotics^14^. Three systems are known to prevent damage resulting from surface exposed phospholipids in Gram negative bacteria, including the phospholipase PldA, the palmitoyltransferase PagP and the VacJ-MlaBCDEF phospholipid transport system^15^. In this study, we elucidate the involvement of VacJ in NTHi respiratory infection, as part of the only system known to maintain the outer membrane lipid asymmetry found to be present in available genomes of this bacterial pathogen, likely to be part of its core genome. Based on the absence of gene homologs, our observations are likely to be unrelated to a concerted action of VacJ_NTHi_ with PldA. Previous evidence indicates that VacJ is important for NTHi resistance to serum mediated killing via the classical pathway of complement activation^17^, which may relate to *vacJ* gene implication in lung pathogenesis^18^. Here, we link the *vacJ* gene deficiency with increased specific fatty acyl residues in bacterial global fatty acid and phospholipid composition, which may contribute to raise bacterial surface hydrophobicity therefore jeopardizing the outcome of NTHi interaction with hydrophobic and lipophilic molecules. Of note, similar outcomes were observed upon NTHi *vacJ* mutant interaction not only with hydrophobic antibiotics and synthetic antimicrobial peptides, but also with free fatty acids shown to be present in the host lungs, further strengthening the importance of VacJ to maintain NTHi membrane stability during its persistent interplay with the host at the human airways.

Fatty acid composition has been previously determined for *H. influenzae* RdKW20 purified outer membrane vesicles and outer membranes, showing abundance of myristic, palmitic, palmitoleic and stearic acid^43,44^. To our knowledge, this is the first report on NTHi total fatty acid composition, further determined in two genetically different strains. Our findings support previous observations on major fatty acyl species, and also highlight the presence of hydroxymyristic acid. This fatty acid is present in the lipid A molecule^24^, but absent in total, outer membrane vesicle and outer membrane phospholipids of NTHi strains (Fig. 2 and^43^), therefore narrowing its location at the lipid A. Likewise, phospholipid content and species composition in the PE fraction has been previously determined in NTHi purified outer membrane vesicles and outer membranes^43,44^. Here, we report NTHi phospholipid total content and fatty acyl composition, and show palmitic and stearic acid as shared species in both PG and PE. *vacJ* deletion has been shown to result in the asymmetric expansion of the outer leaflet, supporting the outward budding of the outer membrane to finally form outer membrane vesicles enriched in phospholipids with decreased palmitic and increased myristic acid amounts^43^. Our results showed that *vacJ* mutation increases total fatty acid content, and *vacJ* deficient strains showed higher amount of fatty acyl residues, including palmitic acid.

Besides VacJ involvement in NTHi membrane integrity and serum resistance^17^, it also contributes maintaining *E. coli*, *Actinobacillus pleuropneumoniae*, *Haemophilus parasuis* and *Shigella flexneri* membrane integrity^15,20,21,23^, *A. pleuropneumoniae* and *H. parasuis* serum resistance and biofilm formation^20,23^, and *H. parasuis* virulence *in vivo*^20^. Moreover, VacJ participates in *H. parasuis* epithelial adhesion and invasion, and in *S. flexneri* cell to cell spread^20,21^. In this study, we show that VacJ plays a role in NTHi airway epithelial infection, although a direct role for VacJ as a bacterial ligand could not be established. Next to chromosome complementation, the *vacJ*_NTHi375_ gene HA-tagged at the 3′ end, together with its predicted promoter region, was cloned and expressed in *E. coli* as a heterologous host; despite VacJ_NTHi375_-HA expression, confirmed by western blot using a monoclonal mouse anti-HA antibody, infection of A549 cells was similar to that shown by control *E. coli* strain harbouring the empty plasmid (Fig. S4). We cannot exclude that defective cell infection could be an indirect effect of *vacJ* disruption, causing outer membrane alterations not only in phospholipids and fatty acids but also in bacterial ligands, which may altogether hinder complementation. The reason for this unsuccessful complementation is currently unknown. Operon prediction by using the MicrobesOnline tool^45^ showed that the *vacJ* gene is unlikely to be part of an operon in *H. influenzae*, therefore excluding possible polar effects. In fact, phenotypic restoration was successful when testing bacterial deoxycholate survival and ChoP levels, and partial when testing fatty acid composition and fatty acid survival, further supporting the complementation approach employed in this study.

LOS decoration with ChoP is known to relate to NTHi resistance to antimicrobial peptides^46^. Unexpectedly, *vacJ* gene deficiency was shown to increase NTHi ChoP levels (Fig. 1F and^17^) but also antimicrobial susceptibility, therefore excluding a VacJ-mediated relationship between bacterial ChoP decoration and resistance to PxE. This observation could relate to the lower intensity of the ion peak representing the monophosphorylated hexa-acylated lipid A observed in the *vacJ* mutant strains (Fig. 2B). Likewise, the *vacJ* gene is known to play a role in resistance to oxidative stress in *Campylobacter jejuni*^47^. Oxidative stress occurs in the small airways, lung parenchyma and alveolar regions in COPD; in asthma, the larger airways are its major site of action. Different to *C. jejuni*, the diameter of NTHi bacterial growth inhibition around H_2_O_2_ soaked discs was comparable between NTHi Δ*vacJ* and WT strains (Fig. S1D). Lastly, this work shows VacJ requirement for NTHi pulmonary infection in mice, supporting previous observations^18^, and NTHi375Δ*vacJ* showed a comparable level of attenuation in mice with either normal lung function or lung emphysema. The panel of fatty acids identified and quantified in murine lung samples, next to other soluble and cellular host factors such as antimicrobial peptides or alveolar macrophages, may contribute to the observed *vacJ* mutant decreased numbers, compared to those by the WT strain. Similar fatty acid composition for both normal function and emphysematous murine lung samples may contribute explaining the comparable *vacJ* mutant loads in the two tested *in vivo* models. We acknowledge that the emphysema model used in this study generated lung lesions (Fig. S3), but did not modify lung fatty acid content, which may relate to potential limitations of the procedure used for emphysema induction over a 17 days time window. Further work will be needed to assess the effect of longer elastase treatment on the murine lung pathophysiology, also considering fatty acid content.

Analysis of *H. influenzae* genome sequences revealed the absence of homologs to *E. coli* PldA, therefore excluding a relationship between *vacJ* disruption and phospholipase-mediated removal of phospholipids at the bacterial surface. Similarly, a homolog to *E. coli* PagP is absent in NTHi, excluding NTHi lipid A hepta-acylation, as shown by lipid A determination for WT and *vacJ* mutant strains. Moreover, *vacJ* disruption did not modify lipid A hexa-acylation, excluding a functional relationship between VacJ and the acyltransferase HtrB. Analysis of *H. influenzae* genome sequences revealed a homolog to the recently characterized *E. coli pqiB* gene, encoding a multi Mammalian Cell Entry (MCE) domain-containing protein displaying a syringe-like architecture periplasmic bridge, which may be involved in maintaining cell envelope homeostasis^48,49^, and will be subject of future study. *H. influenzae* genome sequences also revealed homologs to the *E. coli fadL* and *fadD* genes, annotated as an exogenous long-chain fatty acid transporter and an acyl-CoA synthase/fatty acid-CoA ligase^11,19^, but they lack the *fadE*, *fadB*, *fadH* and *fadA* genes, encoding four enzymes involved in fatty acid degradation via β-oxidation^50^, and genes encoding enzymes of the so called β-oxidation complex II^51^. Conversely, NTHi contains the type II fatty acid synthesis (FASII) system, only lacking the *fabF* gene^50,52,53^, whose products are substrates for acyltransferases catalysing the initial steps in the biosynthesis of phospholipids and lipid A. Those acyltransferases encoding genes (*plsB* and *plsC* for phospholipids; *lpxA*, *lpxD* and *msbB* for lipid A) are also present in NTHi genomes^11,19^. Based on this gene distribution (for a summary, see Table S3), NTHi may transport long-chain fatty acids to be converted to acyl-CoAs, which could contribute to phospholipid synthesis together with FASII-derived acyl-ACPs, but is unlikely to use such fatty acids as carbon and energy source. In fact, when using arachidonic acid as a carbon source, NTHi did not grow in a chemically defined medium but, in turn, its survival was reduced in a VacJ-dependent manner. Same observations were made for oleic acid, supporting the notion that free fatty acid bactericidal effect may be a selective pressure for NTHi to develop counteracting adaptive strategies at the human airways during chronic infection. Interestingly, *Helicobacter pylori* is also susceptible to free fatty acids, lacks β-oxidation, and actively exchanges genetic material by natural competence and homologous recombination^54–57^. Despite occupying separate niches at the human body, both pathogens could develop common adaptive strategies contributing to colonization linked to chronic disease conditions. Identification and characterization of such selective pressures and patho-adaptive counteracting mechanisms will be subject of future studies.

## Methods

### Bacterial strains, plasmids, media and growth conditions

NTHi strains were grown at 37 °C, 5% CO_2_ on chocolate agar (Biomérieux) or brain-heart infusion (BHI) agar supplemented with 10 µg/ml hemin and 10 µg/ml nicotinamide adenine dinucleotide (NAD), referred to as sBHI. NTHi liquid cultures were grown in sBHI at 37 °C, 5% CO_2_. Alternatively, NTHi cultures were grown in a defined minimal medium free of fatty acids (MM-FFA) (Table S2). MM-FFA consists of MIV solutions S21, S22, S23 and S24 (100:1:1:1)^58^, 10 µg/ml hemin and 10 µg/ml NAD. When necessary, MM-FFA was supplemented with (i) glucose 20 mM, (ii) a free fatty acid, (iii) a free fatty acid vehicle solution. Tested free fatty acids were arachidonic and oleic acid, purchased from Sigma-Aldrich. Spectinomycin 30 µg/ml (Spec_30_) and erythromycin 11 µg/ml (Erm_11_) were used when required. *Escherichia coli* was grown on Luria Bertani (LB) broth or LB agar at 30 °C or 37 °C, supplemented with ampicillin 100 µg/ml (Amp_100_), spectinomycin 50 µg/ml (Spec_50_) or erythromycin 150 µg/ml (Erm_150_), when necessary.

For *vacJ* gene disruption, a DNA fragment containing the *vacJ* gene and its respective adjacent regions (1,907 bp) was PCR amplified using genomic DNA from NTHi375 as a template and primers vacJ-F1/931 (5-ATGACTGAAGAAACAACAGTATCAAAA) and vacJ-R1/933 (5′-TTATTTTCTCACTGCAATCGCTTCAATTTC). The gene containing PCR product was cloned into pGEM-T easy (Promega), generating pGEMT-*vacJ*. A Spec^r^ cassette was PCR amplified from pRSM2832 using gene-specific mutagenic primers vacJ-F2/1354 (5′-TTTCACAAATTAGGTAGAATAATCCCATATTTATGTAAGGATCAAAAATGATTCCGGGGATCCGTCGACC) and vacJ-R2/1355 (5′-TTAATTGGCGTGGTGTTGTTGCATTATAAATCGGTAGCACAATATAAGTGTGTAGGCTGGAGCTGCTTCG)^59^. Primers were designed to delete sequences between the start codon and the last 7 codons of *vacJ*. *E. coli* SW102 cells were prepared for recombineering, co-electroporated with pGEMT-*vacJ* (Amp^r^) (50 ng) and the *vacJ*-specific mutagenic cassette (Spec^r^) (200 ng)^60^. Mutagenized clones containing pGEMT-*vacJ::spec* were selected on LB agar with Amp_100_, Spec_50_. This plasmid was used as a template to amplify the *vacJ::spec* disruption cassette with primers vacJ-F1/931 and vacJ-R1/933, which was used to transform NTHi375 and RdKW20 using the MIV method^58^. Transformants were selected on sBHI agar with Spec_30_, to obtain NTHi375Δ*vacJ* and RdKW20Δ*vacJ* mutants. Disruptions were confirmed by PCR. Complementation of NTHi375Δ*vacJ* and RdKW20Δ*vacJ* mutant strains was performed by inserting the *vacJ* gene into open reading frame (ORF) HI0601.1; this ORF contains a frameshift in both RdKW20 and NTHi375^31^. PCR was used to amplify a 2,342 kb fragment from NTHi375 genomic DNA using primers HI0601.1-F1/488 (5′-GAAGTAAGAGATGAAAAGCGAAG) and HI0601.1-R1/489 (5′-TTGGTAAAAATGGATGAAGGGGATTAC). This fragment was cloned into *Sma*I digested pUC19, generating pUC19-HI0601.1, which was used as a template for inverse PCR using primers Hi0601.1up-*Xho*I-R/1493 (5′-CCGCTCGAGACAATCAAACCAATAAACCCGAAA) and Hi0601.1down-*Nco*I-F/1494 (5′-CATGCCATGGGCAGTATTTTAAAAGCGGATATCA). This PCR product was digested with *Xho*I and *Nco*I, used as a vector to clone a *Xho*I and *Nco*I digested Erm resistance cassette which was PCR amplified using pBSLerm^61^ as a template, and primers ermC-*Xho*I-F/1517 (5′-CCGCTCGAGGAGCTCGAATTCGGCTTCATGCT) and ermC-*Nco*I-R/1518 (5′- CATGCCATGGGGTACCGAGCTCGAATTCGGCTTGATCG), to generate pUC19-HI0601.1-Erm. The *vacJ* gene with its promoter region was amplified from NTHi375 genomic DNA using primers vacJ-*Xho*I-F/1523 (5′-CCGCTCGAGATGACTGAAGAAACAACAGTATCAAAA) and Pr-vacJ-HA-*Xho*l-R/1524 (5′-CCGCTCGAGTTAAGCGTAGTCTGGGACGTCGT). This PCR product (1,487 bp) was *Xho*I digested and cloned into *Xho*I-digested pUC19-HI0601.1-Erm, generating pUC19-HI0601.1-Erm-*Pr::vacJ*. This plasmid was used as a template to amplify a 5,015 kb DNA fragment containing HI0601.1 flanked *Pr::vacJ*, used to naturally transform NTHi375Δ*vacJ* and RdKW20Δ*vacJ* strains. Complemented strains were selected on sBHI agar with Spec_30_ and Erm_11_.

### Prediction of VacJ_NTHi_ characteristics and lipobox motif

Subcellular location of the VacJ lipoprotein was predicted using the Cell-Ploc package (http://chou.med.harvard.edu/bioinf/Cell-PLoc/)^62^. VacJ characteristics were predicted with the PROTEAN program and proteomics tools from the ExPASy website. VacJ lipobox sequence was predicted using the DOLOP program^63^.

### Bacterial growth

Strains were grown on chocolate agar for 16 h. Bacterial suspensions collected in PBS were normalized to OD_600_ = 1, diluted to OD_600_ = 0.05 in sBHI, and 200 µl aliquots were transferred to individual wells in 96-well microtiter plates (Falcon). Plates were incubated with agitation at 37 °C for 12 h in a Multiskan instrument (Thermo Scientific), and OD_636_ was monitored every 15 min. Each growth curve was corrected to its blank values (sBHI). Alternatively, a bacterial suspension recovered with 1 ml MM-FFA from a freshly grown chocolate agar plate was adjusted to OD_600_ = 1. Arachidonic acid stock solution (100 mM) was prepared and diluted to the requiredworking concentrations in ethanol. Then, 160 μl of (i) MM-FFA, (ii) MM-FFA with arachidonic acid 25 μM, (iii) MM-FFA with fatty acid vehicle solution, i.e. ethanol volumes identical to those used for arachidonic acid 25 μM, (iv) MM-FFA with glucose 20 mM, were transferred to individual wells in 96-well microtiter plates (Sarstedt). Next, 40 μl of the previously prepared bacterial suspensions were added to each well. Plates were incubated in a SpectraMAX 340 microplate reader at 37 °C, and OD_600_ was recorded every 30 min for 12 h.

### Lipid A purification and analysis

Lipid A was extracted and processed by using an ammonium hydroxide/isobutyric acid method and subjected to negative-ion matrix-assisted laser desorption ionization time-of-flight (MALDI-TOF) mass spectrometry^25^. Freshly grown bacteria on chocolate agar were scraped from the plates, resuspended in 400 µl isobutyric acid–1 M ammonium hydroxide (5:3 [v/v]) and incubated in a screw-cap test tube at 100 °C for 2 h, with occasional vortexing. Samples were cooled in ice water and centrifuged (2,000 × *g* for 15 min). The supernatant was transferred to a new tube, diluted with an equal volume of water, and lyophilized. The sample was then washed twice with 400 µl methanol and centrifuged (2,000 × *g* for 15 min). The insoluble lipid A was solubilized in 100 to 200 µl chloroform-methanol-water (3:1.5:0.25 [v/v/v]). Analyses were performed on a Bruker autoflex® speed TOF/TOF mass spectrometer (Bruker Daltonics Inc.) in negative reflective mode with delayed extraction. The ion-accelerating voltage was set at 20 kV. To analyse the samples, 1 to 3 µl of lipid A suspension (1 mg/ml) were desalted with a few grains of ion-exchange resin (Dowex 50W-X8; H) in a 1.5 ml microcentrifuge tube. A 1 µl aliquot of the suspension (50 to 100 µl) was deposited on the target and covered with the same amount of dihydroxybenzoic acid matrix (Sigma-Aldrich) dissolved in 0.1 M citric acid. Different ratios between the samples and dihydroxybenzoic acid were used when necessary. Alternatively, lipid A was mixed with 5-chloro-2-mercapto-benzothiazole (Sigma-Aldrich) at 20 mg/ml in chloroform-methanol (1:1 [v/v]) at a ratio of 1:5. Each spectrum was an average of 300 shots. A peptide calibration standard (Bruker Daltonics) was used to calibrate the MALDI-TOF. Further calibration for lipid A analysis was performed externally using lipid A extracted from *E. coli* strain MG1655. Interpretation of the negative-ion spectra was based on earlier studies showing that ions with masses higher than 1,000 gave signals proportional to the corresponding lipid A species present in the preparation^64^.

### NTHi lipid extraction and phospholipid separation by TLC

Bacterial total lipids were extracted using the Bligh and Dyer Method^65^. Briefly, 10 ml of bacterial suspensions normalized in distilled water (OD_600_ = 1) were prepared by collecting biomass from NTHi strains grown on chocolate agar. Bacterial suspensions were pelleted, resuspended in 0.2 ml distilled water and 0.75 ml chloroform:methanol (1:2 [v/v]), incubated for 5 min with- and for 15 min without shaking, followed by centrifugation (15,000 *g*, 20 min). Supernatants were transferred to a new tube with 0.5 ml chloroform:water (1:1 [v/v]), agitated for 5–15 min, and centrifuged (15,000 *g*, 20 min) to separate organic and aqueous phases. Next, 0.4 ml of the organic phase (bottom) were transferred to a clean tube. All steps were performed at room temperature (RT). Chloroform was evaporated from samples (SpeedVac) and dried extracts were stored at −20 °C prior TLC. Dried lipids were dissolved in 60 μl chloroform and spotted in duplicate onto a TLC plate (silica gel 60, Merck). TLC plates were placed in a solvent saturated tank (chloroform:methanol:acetic acid, 13:5:2 [v/v/v]) for phospholipid separation. After separation, dried TLC plates were placed in an iodine chamber for band visualization, scraped or, alternatively, developed by charring with sulfuric acid 15% in ethanol at 180 °C.

### Determination fatty acid composition

Fatty acid composition and quantity were determined by GC-MS of the corresponding fatty acid methyl esters (FAMEs). NTHi FAMES were prepared as it follows: triplicates of chocolate agar grown bacteria were normalized in 1 ml PBS to OD_600_ = 3 and pelleted to obtain ∼40 mg bacterial biomass. Both these samples and scraped phospholipids from TLC separation (see section above), were treated with NaOH 15% (1 ml) in methanol:water (1:1, v/v) for 30 min at 100 °C. Saponified material was methylated with methanol (2 ml) in 50% (v/v) HCl 6 N for 10 min at 80 °C. In this step, 20 μl of the internal standard C17:0 (1 mg/ml) were added. NTHi FAMEs were extracted with 1.25 ml hexane:methyl tertbutyl ether (1:1, v/v), and added to a new tube (900 μl) containing 4 ml NaOH 3% in distilled water as washing step. ∼300 μl of remaining the organic phase (top) were carefully transferred to a GC vial and capped (Technical note #101, MIDI). For murine lung FAMEs preparation, lung homogenates (see below) were stored at −80 °C. Five hundred μl of each homogenate were mixed with 750 μl HCl-methanol (3 N), 150 μl toluene and 20 μl of the GC-MS internal standard (C17:0, 1 mg/ml), and incubated at 90 °C for 1 h. Next, 500 µl NaCl 0.9% and 500 µl hexane were added, mixed vigorously, and centrifuged at 2,000 r.p.m for 5 min at RT. Supernatants (~300 µl) were transferred to a new tube, dried overnight in a chemical cabinet at RT, and the pellet obtained was resuspended in 100 µl hexane for further GC-MS analysis.

GC-MS analyses of bacterial and murine lung FAMEs were performed using a 7890A GC device coupled to a 5975C Inert XL MSD mass selective detector (Agilent Technologies, Santa Clara, USA). A volume of 1 μl was injected on an Agilent J&W DB-WAX column (diameter, 0.25 mm; film thickness, 0.25 μm; length, 30 m) with a 1 ml/min helium flow. Injection parameters were as it follows: split mode injection (1:10) and injector temperature 250 °C. The temperature gradient was 1 min at 50 °C; 25 °C/min up to 200 °C; 3 °C/min up to 230 °C and then 18 min at 230 °C, and the solvent delay was 5 min. The source was set to 230 °C and 70 eV, scanning at 20 scans/min, from 40 to 500 m/z. FAMEs were identified by comparing their mass spectra with those of the NIST library and by comparison of the retention times with a FAME standard mix (Sigma-Aldrich).

### Bacterial hydrophobicity

Duplicates of chocolate agar grown bacteria were normalized in 2.5 ml PBS to OD_600_ = 0.5 (A_1_) in capped tubes. Next, 0.5 ml xylene (PanReac AppliChem) were added per tube, the mixture was incubated at 44 °C for 10 min, vigorously vortexed for 1 min, incubated under the same conditions for 1 h, and the aqueous phase OD_600_ (A_2_) was measured. Results are expressed as percentage of bacterial surface hydrophobicity ([(A_1_ − A_2_)/A_1_] × 100). Strong hydrophobic and hydrophilic bacteria get values >50% and <20%, respectively^66^.

### Antimicrobial susceptibility testing

Susceptibility to ampicillin, amoxicillin-clavulanic acid, cefuroxime, cefepime, cefotaxime, ceftriaxone, imipenem, meropenem, chloramphenicol, tetracycline, ciprofloxacin, cotrimoxazole and azithromycin was determined by microdilution according to the criteria of the Clinical Laboratory Standards Institute (CLSI)^67^. Susceptibility to the hydrophobic antibiotics erythromycin, azithromycin and rifampicin was also determined by E-test (Biomérieux) and/or disc diffusion (Becton Dickinson). For PxE, bacterial susceptibility was determined by E-test (Biomérieux).

### Detergent susceptibility testing

PBS normalized bacterial suspensions (OD_600_ = 1) were prepared by using NTHi strains freshly grown on chocolate agar. Then, 100 µl of normalized suspensions were transferred to individual wells in 96-well microtiter plates (Sarstedt), to be incubated with 100 µl sodium deoxycholate (Alfa-Aesar) for 20 min at RT in static conditions. A deoxycholate stock solution (43.25 mg/ml) was prepared in dH_2_O and diluted to the required working concentrations in PBS (0.5; 0.75; 1; 1.25; 1.5 mg/ml). Bacterial suspensions were next used to (i) measure OD_405_; (ii) ten-fold dilution in PBS and plating on sBHI agar for c.f.u. counting (represented as log_10_ c.f.u./ml); (iii) five µl of serial culture dilutions (10^−1^) from each well were spotted on sBHI agar for visualization.

### TEM

PBS normalized NTHi suspensions (OD_600_ = 1) were prepared by using NTHi strains freshly grown on chocolate agar. Then, 100 µl of such suspensions were incubated with none or with 100 µl sodium deoxycholate 0.5 mg/ml for 20 min at RT in static conditions. Bacteria were next applied to Formvar-coated grids, air dried, negatively stained with 1% phosphotungstic acid in distilled water for 10 s, and examined with a JEM-1011 transmission electron microscope (JEOL) operating at 80 kV and equipped with an Orius SC1000 charge-coupled device (CCD) camera (Gatan).

### Free fatty acids susceptibility testing

MM-FFA normalized bacterial suspensions (OD_600_ = 0.1) were prepared by using NTHi strains grown on chocolate agar. Oleic and arachidonic acid stock solutions (100 mM) were prepared and diluted to the required working concentrations in ethanol. For arachidonic acid, tested concentrations ranged from 3 to 75 μM; for oleic acid, tested concentrations ranged from 0.8 to 2 mM. MM-FFA with fatty acid (160 µl) was transferred to individual wells in 96-well microtiter plates (Sarstedt); 40 µl of the previously prepared bacterial suspensions were added to each well, and incubated for 20 h at 37 °C with 5% CO_2_ in static conditions. Vehicle solution, consisting of an ethanol volume equivalent to that used for the highest fatty acid concentration tested, and only MM-FFA controls were performed in parallel. After incubation, bacteria were serially diluted in PBS and plated on sBHI agar. Results are expressed as percentage of bacterial survival ([c.f.u.ml^−1^_fatty acid_ × 100]/c.f.u.ml^−1^_vehicle_).

### ChoP quantification

Three to four colonies of NTHi grown on chocolate agar were inoculated into 20 ml sBHI, grown for 11 h, diluted in 40 ml sBHI to OD_600_ = 0.05, grown to OD_600_ = 0.6, serially diluted, plated on sBHI agar for c.f.u. determination, and used to generate stocks stored at −80 °C in sBHI with 20% glycerol as single use aliquots. An aliquot containing ~10^7^ c.f.u. was thawed and incubated for 1 h at 37 °C with TEPC-15, a mouse monoclonal antibody specific for ChoP (Sigma-Aldrich) diluted 1:25 in PBS-0.05% Tween 20. Samples were washed twice with PBS-0.05% Tween 20, and incubated with a fluorescein isothiocyanate (FITC)-conjugated rabbit anti-mouse (Serotec) diluted 1:300 in PBS-0.05% Tween 20 for 30 min at 4 °C under dark conditions. Bacteria were washed with PBS-0.05% Tween 20, fixed in 3% paraformaldehyde (PFA) for 2–3 min at RT, and analysed on a FACSCalibur flow cytometer (BD Biosciences) using forward and side scatter parameters to gate on at least 25,000 bacteria. Results are expressed as a relative percentage fluorescence index (RFI), to measure both the proportion of fluorescent bacteria positive for ChoP and the intensity of fluorescence^34^.

### RNA extraction and RT-qPCR analysis

NTHi strains were grown on chocolate agar. Bacteria (2 to 5 colonies) were inoculated into 20 ml sBHI, grown for 11 h, diluted into 40 ml fresh sBHI to OD_600_ = 0.05, and grown to OD_600_ = 0.6. Bacterial total RNA was isolated using TRIzol reagent (Invitrogen), according to manufacturer’s instructions. Total RNA quality was evaluated using RNA 6000 Nano LabChips (Agilent 2100 Bioanalyzer). All samples had intact 16 S and 23 S ribosomal RNA bands. Reverse transcription was performed using 1 µg RNA by PrimerScript RT Reagent kit (Takara). To monitor *lic1A* gene expression, PCR amplification was performed by using SYBR Premix Ex Taq II (Tli RNaseH Plus) (Takara) and primers Lic1ART-Fw/1098 (5′-TCCATTCCTTTTTCCTCCATTG) and Lic1ART-Rv/1099 (5′-AAGTAGAACATTTTGATTGGTCATTCC). 16S ribosomal RNA was amplified with primers 16S-Fw/1074 (5′-GGCGTTGATGACCGTGAAAC) and 16S-Rv/1075 (5′-GCCAGTAATAATCGCCCTCTTCTAG). Fluorescence data were analysed with AriaMx Real-Time PCR System (Agilent Technologies). Relative mRNAs quantities were calculated using the comparative threshold cycle (Ct) method and normalized using 16S ribosomal RNA (16SrRNA) as an endogenous control.

### Cell culture and bacterial infection

A549 human alveolar basal epithelial cells (ATCC CCL-185) were maintained and seeded as described^68^. NCI-H292 mucoepidermoid pulmonary human carcinoma epithelial cells (ATCC CRL-1848) were maintained and seeded as described^29^. Invasion assays were performed and processed as described^29,30,32,33,68^. For infection, PBS-normalized bacterial suspensions (OD_600_ = 1) were prepared by using NTHi strains grown on chocolate agar. A multiplicity of infection (MOI) of ~100:1 was used. Cells were infected for 2 h in 1 ml EBSS (Earle’s Balanced Salt Solution, Gibco), washed 3 times with PBS, incubated for 1 h with RPMI 1640 medium containing 10% FCS, Hepes 10 mM and gentamicin 200 μg/ml. Cells were then washed 3 times with PBS, lysed with 300 μl PBS-saponin 0.025% for 10 min at RT, and serial dilutions were plated on sBHI agar. Results are expressed as c.f.u./well.

### Secretion of IL-8

Bacteria grown on chocolate agar were collected with PBS, suspensions were normalized to OD_600_ = 1, and used for 2 h infections. A549 cells were washed 3 times with PBS, and incubated for 6 h in RPMI 1640 medium containing 10% FCS, Hepes 10 mM and gentamicin 100 µg/ml. Supernatants were collected from the wells, cell debris removed by centrifugation and samples frozen at −80 °C. IL-8 levels in the supernatants were measured by ELISA (Abnova KA0115) with sensitivity <2 pg/ml. Results are expressed as IL-8 pg/ml.

### NTHi mouse lung infection

A CD1 mouse model of NTHi lung infection was used^29,32–34^. CD1 female mice (18–20 g) aged 4 to 5 weeks purchased from Charles River Laboratories (France) were housed under pathogen-free conditions at the Institute of Agrobiotechnology facilities (registration number ES/31-2016-000002-CR-SU-US). Animal handling and procedures were in accordance with the current European (Directive 86/609/EEC) and National (Real Decreto 53/2013) legislations, following the FELASA and ARRIVE guidelines, and with the approval of the Universidad Pública de Navarra (UPNa) Animal Experimentation Committee (Comité de Ética, Experimentación Animal y Bioseguridad) and the local Government authorization. NTHi375 WT and mutant strains were used for lung infection. When necessary, mice were randomly divided into three groups (n ≤ 8): (i) control mice with normal lung function; (ii) mice with lung emphysema; (iii) elastase vehicle solution. Emphysema was induced by intratracheal administration of porcine pancreatic elastase (EPC, Elastin Products Company). To do so, 10 mg containing 1,350 elastase units (U) were resuspended in 10 ml physiological serum to generate a stock solution (1 mg/ml, i.e. 135 U/ml). To induce emphysema, one 90 μl dose containing 6 elastase U/mouse was administered 17 days before infection. Infecting bacteria were previously grown on chocolate agar. For NTHi intranasal infection, 20 μl of a bacterial suspension containing ~2 × 10^8^ c.f.u. was placed at the entrance of the nostrils until complete inhalation by each mouse, previously anesthetized (ketamine-xylazine, 3:1). When indicated, mice were euthanized and lungs aseptically removed. The left lung was individually weighed in sterile bags (Stomacher80, Seward Medical) and homogenized 1:10 (w/v) in PBS. Each homogenate was serially 10-fold diluted in PBS and plated in triplicate on sBHI agar to determine the number of viable bacteria. Results are shown as log_10_ c.f.u./lung. When required, remaining homogenate material was stored at −80 °C to determine lung fatty acid composition (see section above). In parallel, BALF samples were obtained in animals with normal lung function by perfusion and collection of 0.7 ml of PBS, with the help of a sterile 20 G (1.1-mm diameter) Vialon intravenous catheter (Becton-Dickinson) inserted into the trachea. Each recovered BALF fraction was serially 10-fold diluted and plated on sBHI agar. Results are shown as log_10_ c.f.u./ml BALF.

### Animal preparation and pulmonary function tests (PFT)

Pulmonary function tests were performed in all animals before micro-CT acquisition. To this end, animals were first anesthetized with an intraperitoneal injection of 90 mg/kg ketamine (Imalgene®, Merial, France) and 10 mg/kg xylazine (Rompun®, Bayer AG, Germany). Anesthetized animals were intratracheally cannulated and connected to a Flexivent rodent ventilator (Scireq, Montreal, Canada) set at a rate of 200 breaths/min and a tidal volume of 10 ml/kg. Animals were kept breathing isoflurane at 2% concentration until completely relaxed. Lung resistance (R, measured in cmH_2_O.s/mL), compliance (C, measured in mL/cmH_2_O) and elastance (E, measured in cmH_2_O.s/mL) parameters were measured by using a single-frequency-forced oscillation, fitting the measured data to a single compartment model of the lung. All measurements were repeated at least five times.

### Breath-hold gated micro-CT imaging and image analysis

Lung 3D tomographic images were acquired using X-ray micro-CT (Micro-CAT II, Siemens PreClinical Solutions, Knoxville, Tennessee) with the following parameters: 80 kVp X-ray source voltage, 500 μA current and 450 ms exposure time per projection. Seven hundred micro-CT projections were acquired during 650 ms iso-pressure breath holds at 12 cm H_2_O. Normal breathing was induced during two complete respiratory cycles between breath holds. A total lung capacity perturbation was performed every 20 breath holds to prevent atelectasis. The tomographic three-dimensional images obtained had a total of 640 slices with isotropic 46 μm voxel size and a resolution of 1024 × 1024 pixels per slice. Micro-CT images were automatically reconstructed by using the Cobra software (Exxim Computing Corporation, CA, USA) and 3D lung images were processed by using the Amira 3D Software for preclinical analysis (Thermo Scientific, MA, USA).

### Statistical analysis

In all cases, p < 0.05 value was considered statistically significant. Analyses were performed using Prism software, version 7 for Mac (GraphPad Software) statistical package.

## Electronic supplementary material

Supplementary Material
