# Supplementary material for: Modulation of Haemophilus influenzae interaction with hydrophobic molecules by the VacJ/MlaA lipoprotein impacts strongly on its interplay with the airways
Source: Sci Rep. 2018 May 2;8:6872. doi: 10.1038/s41598-018-25232-y (PMC5932069; doi:10.1038/s41598-018-25232-y)
Supplement: Supplementary file 1 — Supplementary Material [file 41598_2018_25232_MOESM1_ESM.pdf]

**Supplementary Material**

# Modulation of *Haemophilus influenzae* interaction with hydrophobic molecules by the VacJ/MlaA lipoprotein impacts strongly on its interplay with the airways

Ariadna Fernández-Calvet[1], Irene Rodríguez-Arce[1], Goizeder Almagro[1], Javier Moleres[1], Begoña Euba[1,2], Lucía Caballero[1], Sara Martí[2,3], José Ramos-Vivas[4,5], Toby Leigh Bartholomew[6], Xabier Morales[7], Carlos Ortíz-de-Solórzano[7,8], José Enrique Yuste[2,9], José Antonio Bengoechea[6], Raquel Conde-Álvarez[10], Junkal Garmendia[1,2]

[1]Instituto de Agrobiotecnología, CSIC-Universidad Pública Navarra-Gobierno Navarra, Spain; [2]Centro de Investigación Biomédica en Red de Enfermedades Respiratorias (CIBERES), Madrid, Spain; [3]Departamento Microbiología, Hospital Universitari Bellvitge, University of Barcelona, IDIBELL, Barcelona, Spain; [4]Servicio Microbiología, Hospital Universitario Marqués de Valdecilla and Instituto de Investigación Marqués de Valdecilla (IDIVAL), Santander, Spain; [5]Red Española de Investigación en Patología Infecciosa (REIPI), ISCIII, Madrid, Spain; [6]Centre for Experimental Medicine, Queen's University Belfast, Belfast, UK; [7]Laboratory of Preclinical Models and Analytical Tools, Division of Solid Tumors and Biomarkers, Center for Applied Medical Research, Pamplona, Spain; [8]Centro de Investigación Biomédica en Red de Enfermedades Oncológicas (CIBERONC), Madrid, Spain; [9]Centro Nacional de Microbiología, Instituto de Salud Carlos III (ISCIII), Madrid, Spain; [10]Instituto de Salud Tropical, Instituto de Investigación Sanitaria de Navarra and Dpto. Microbiología y Parasitología, Universidad de Navarra, Edificio de Investigación, Pamplona, Spain

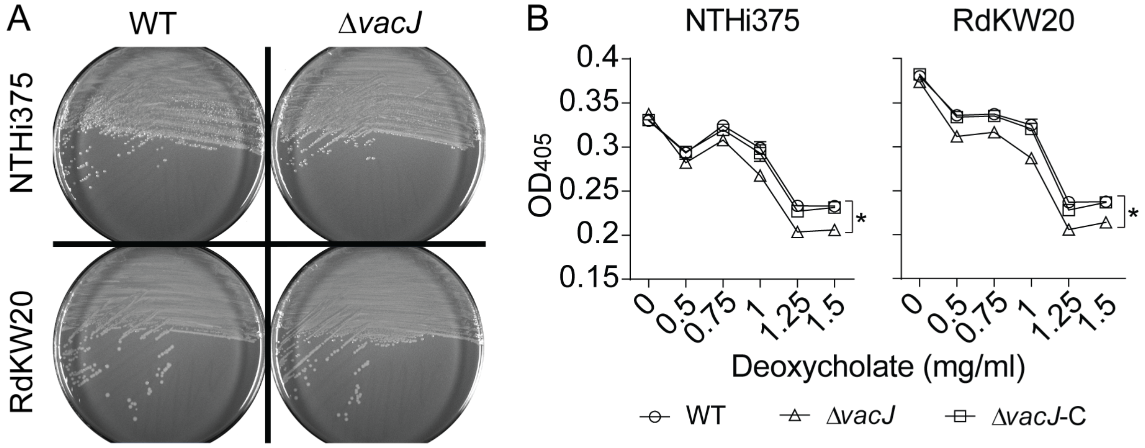


C CLUSTAL O(1.2.4) multiple sequence alignment

```
NTHi375          ATGAATACAAAAATGCTATGCAATCAATCAATCAATCAATCAATCAATCAATCAATCAAT
NTHi375ΔvacJ     ATGAATACAAAAATGCTATGCAATCAATCAATCAATCAATCAATCAATCAATCAATCAAT
RdKW20           ATGAATACAAAAATGCTATGCAATCAATCAATCAAT--------------------CAAT
RdKW20ΔvacJ      ATGAATACAAAAATGCTATGCAATCAATCAATCAAT--------------------CAAT
                 ************************************                    ****

NTHi375          CAATCAATCAATCAATCAATCAATCAATCAATCAATCAATCAATCAAATTGTAGGATTTG
NTHi375ΔvacJ     CAATCAATCAATCAATCAATCAATCAATCAATCAATCAATCAATCAAATTGTAGGATTTG
RdKW20           CAATCAATCAATCAATCAATCAATCAATCAATCAATCAATCAATCAAATTGTAGGATTTG
RdKW20ΔvacJ      CAATCAATCAATCAATCAATCAATCAATCAATCAATCAATCAATCAAATTGTAGGATTTG
                 ************************************************************

NTHi375          TTAAAACTTGCT
NTHi375ΔvacJ     TTAAAACTTGCT
RdKW20           TTAAAACTTGCT
RdKW20ΔvacJ      TTAAAACTTGCT
                 ************
```

D

| | NTHi375 | | RdKW20 | |
|---|---|---|---|---|
| $H_2O_2$ (μmol) | WT | Δ*vacJ* | WT | Δ*vacJ* |
| 3.15 | 1.68 ± 0.1 | 1.70 ± 0.1 | 0.67 ± 0.1 | 0.63 ± 0.1 |
| 6.25 | 2.07 ± 0.2 | 2.07 ± 0.2 | 0.80 | 0.77 ± 0.1 |
| 12.5 | 2.48 ± 0.2 | 2.47 ± 0.2 | 1.00 | 1.00 |
| 25 | 2.88 ± 0.3 | 2.97 ± 0.4 | 1.43 ± 0.1 | 1.30 |

**Figure S1. (A)** Morphology of bacterial colonies rendered by NTHi375 and RdKW20 WT and *vacJ* mutant strains, grown on chocolate agar for 16 h at 37ºC with 5% $CO_2$. Colony size and morphology was comparable between WT and mutant strains. **(B)** Sodium deoxycholate bactericidal effect on NTHi depends on VacJ. NTHi WT and Δ*vacJ* strains grown on chocolate agar were used to generate $OD_{600}$-normalized bacterial suspensions for further incubation with sodium deoxycholate. After 20 min, deoxycholate dose dependent bactericidal effect was higher on the Δ*vacJ* mutant than on the WT strains, as measured by assessing turbidity (for NTHi375, $p<0.005$; for RdKW20, $p<0.0005$) (WT strains, circle;

Δ*vacJ* mutants, triangle; Δ*vacJ* complemented strains, square). Phenotypic restoration in Δ*vacJ* complemented strains (Δ*vacJ*-C) could be observed. Experiments were performed in quadruplicate, in three independent occasions (n=12). Results are shown as means ± SE; statistical comparisons were performed with two-way ANOVA (Tukey's multiple comparisons test). **(C)** Multiple sequence alignment of the 5´end of the *lic1A* gene containing $(5'\text{-CAAT})_n$ tetranucleotide repeats. Primers Lic1prF1-HindIII/858 (5'-GCGGATTATTACAATCTATTTAACCTC) and Lic1ART-Rv/1099 (5'-AAGTAGAACATTTTGATTGGTCATTCC) were used for DNA amplification using NTHi375 and RdKW20 WT and *vacJ* mutant strains as template, and Sanger sequencing. The *vacJ* gene inactivation did not vary the number of repeats, i.e. 21 in NTHi375 and 16 in RdKW20 strains. **(D)** Effect of $H_2O_2$ on NTHi growth inhibition, measured by disc diffusion assay. NTHi cells were scraped from freshly grown chocolate agar plates into PBS, and adjusted to $OD_{600}$=1. Normalized suspensions containing ~7x$10^8$ c.f.u./ml were plated on sBHI agar. $H_2O_2$ 9.8 M (Sigma-Aldrich) was used as stock solution to be further diluted in sterile distilled water. Sterile paper discs soaked on 10 µl $H_2O_2$ containing 3.15, 6.25, 12.5 or 25 µmol were located on the sBHI agar and plates were incubated. Results are shown as diameter of bacterial growth inhibition (cm). Experiments were performed three times (n=3). No significant differences were observed between WT and mutant strains by means comparison with two-tail *t*-test.

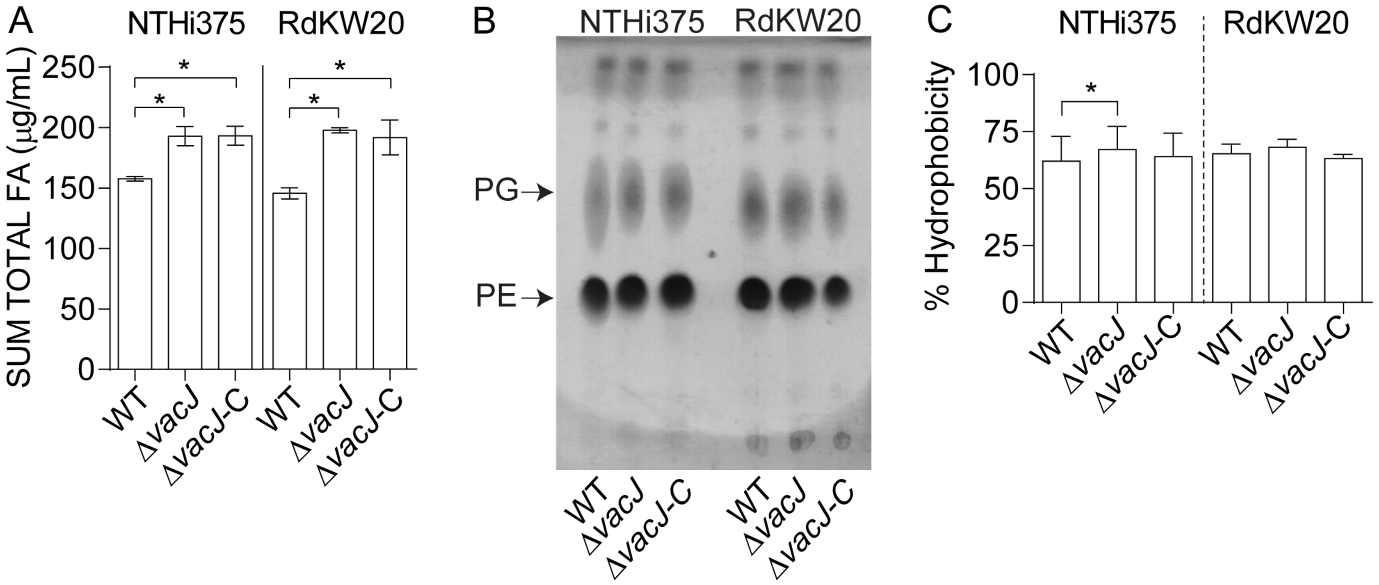


**Figure S2.** Total fatty acids and phospholipids in NTHi WT and *vacJ* mutant strains. **(A)** Total fatty acid composition, as methyl esters (shown as means ± SE). Bacteria grown on chocolate agar were used to extract fatty acids, by following saponification, methylation, extraction and washing steps. *vacJ* mutants showed increased fatty acid amounts (for NTHi375 and RdKW20, p<0.005). Phenotypic alterations were not restored in the *vacJ* complemented strains (Δ*vacJ*-C) (for NTHi375 and RdKW20, *p<0.005). Measures were performed in duplicate, in three independent occasions (n=6). Two-way ANOVA (Sidak's multiple comparisons test) was used for statistical analysis. **(B)** TLC bands corresponding to PE and PG phospholipids, in NTHi375 and RdKW20 WT, Δ*vacJ* and Δ*vacJ* complemented strains. **(C)** Bacterial surface hydrophobicity. Bacteria grown on chocolate agar were processed to measure their hydrophobicity (shown as means ± SD). Bacterial surface hydrophobicity showed a trend to be higher for Δ*vacJ* (NTHi375Δ*vacJ*, 67.2±10.2%; RdKW20Δ*vacJ*, 68.2±3.5%) than for their isogenic WT strains (NTHi375, 62.1±10.9%; RdKW20, 65.4±4.1%). Hydrophobicity rendered values of 64±10.4 and 63.3±1.7% for NTHi375Δ*vacJ*-complemented and RdKW20Δ*vacJ*-complemented strains. Surface hydrophobicity was higher for Δ*vacJ* than for NTHi375 WT strain (*p<0.0005).

Measures were performed in duplicate, in three independent occasions (n=6). Two-way ANOVA (Sidak's multiple comparisons test) was used for statistical analysis.

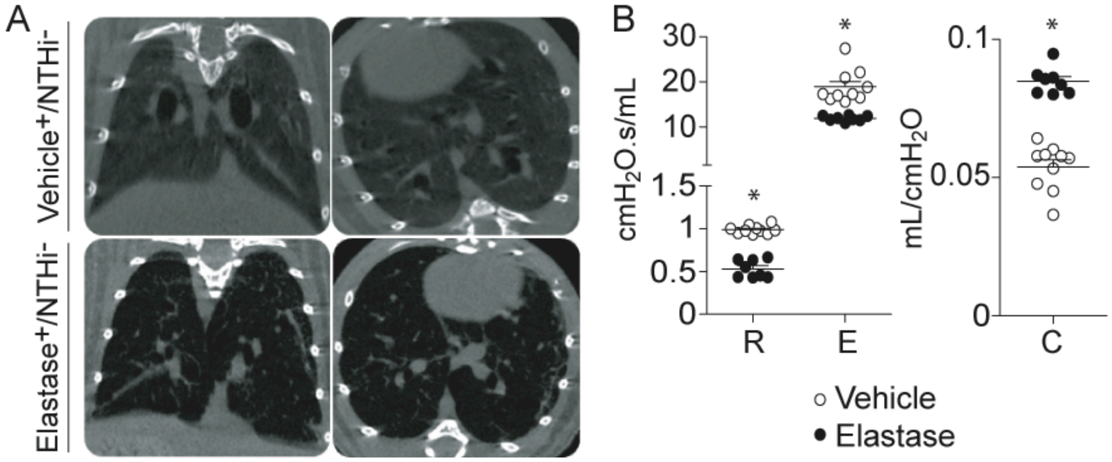


**Figure S3.** Elastase instillation induces a lesion compatible with lung emphysema in CD1 mice (n≥8 per group). Emphysema was induced by intratracheal instillation of 6 U/mouse of porcine pancreatic elastase. In parallel, control animals were instillated with vehicle solution. Lung emphysema was evaluated 17 days after elastase/vehicle administration using X-ray Micro-CT **(A)** and pulmonary function tests (PFT) **(B)**. **(A)** Representative images for longitudinal (left panels) and transversal (right panels) micro-CT slices are shown. Elastase-treated animals presented lower tissue density and abnormally expanded lungs compared to vehicle animals. **(B)** Elastase-treated animals (black circles) showed higher compliance (C), lower resistance (R) and lower elastance (E) than vehicle-treated animals (white circle). Means ± SD are shown, compared by *t*-test, *p<0.0005.

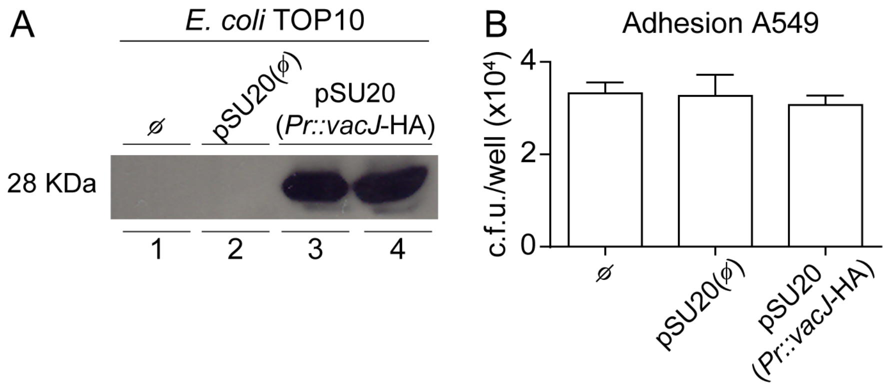


**Figure S4.** Heterologous expression of the *vacJ*$_{\mathrm{NTHi}}$ gene in *E. coli* TOP10 does not modulate bacterial epithelial adhesion. **(A)** The *vacJ*$_{\mathrm{NTHi375}}$ gene with a HA tail in its 3´end, together with its predicted promoter region, were PCR amplified using NTHi375 genomic DNA as template and primers vacJ-F1/931 (5'-ATGACTGAAGAAACAACPAGTATCAAAA) and *Pr-vacJ*-HA-R/1440 (5'-GATGACATACTAAAAGAAATTGATTACCCATACGACGTCCCAGACTACGCTTAA). The 1,487 bp PCR product was cloned into Hinc*II* digested pSU20, generating pSU20-*Pr::vacJ*-HA. VacJ$_{\mathrm{NTHi}}$ protein was monitored in *E. coli* TOP10 by western blot. To this end, whole cell extracts were prepared from bacterial suspensions recovered from LB+Cm 30 μg/ml cultures, adjusted to $OD_{600}$=1 in PBS, lysed by ultrasounds sonication, two-fold diluted with 2X loading buffer (Tris-HCl 62.5mM pH 6.8, SDS 2% w/v, glycerol 10%, DTT 50 mM; Bromophenol Blue 0.01% w/v), and heated to 95ºC for 5 min. Proteins were separated on 12% SDS-PAGE gels (BioRad, MINI PROTEAN Tetra System) and transferred to a nitrocellulose membrane. VacJ protein (~28 KDa) was monitored with primary rabbit anti-HA antibody (Sigma-Aldrich) diluted 1:4000, and secondary goat anti-rabbit IgG (whole molecule, Sigma-Aldrich) antibody conjugated to horseradish peroxidase, diluted 1:1000. Lane 1, untransformed bacteria; lane 2, bacteria transformed with empty pSU20 vector; lanes 3 and 4, bacteria transformed with pSU20-*Pr::vacJ*-HA, two clones were tested. **(B)** A549

cells were infected with *E. coli* TOP10 (MOI~50:1) to quantify adhesion (means ± SD are shown). After infection (30 min at 37ºC and 5% $CO_2$), wells were washed three times with PBS and incubated with saponin 0.025% during 10 min for cell lysis. Lysates were 10-fold serially diluted in PBS and plated on LB agar. Adhesion assays were performed in triplicate, in three independent occasions (n=9). No significant differences were observed (two-tail *t*-test).

**Table S1.** Minimal inhibitory concentration (MIC) of 12 hydrophilic antibiotics against NTHi WT and *vacJ* mutant strains by the microdilution method.

| | **MIC (mg/l)** | | | | | | | | | | | |
|---|---|---|---|---|---|---|---|---|---|---|---|---|
| **NTHi strain** | **Ampicillin** | **Amoxicillin/ Clavulanic** | **Cefuroxime** | **Cefepime** | **Cefotaxime** | **Ceftriaxone** | **Imipenem** | **Meropenem** | **Chloranfenicol** | **Tetracycline** | **Ciprofloxacin** | **Cotrimoxazole** |
| NTHi375 WT | 0,25 | <0,5/0,25 | 2 | <0,25 | <0,06 | <0,12 | 1 | <0,25 | <1 | <1 | <0,03 | <0,5/9,5 |
| NTHi375Δ*vacJ* | 0,25 | <0,5/0,25 | 2 | <0,25 | <0,06 | <0,12 | 0,5 | <0,25 | <1 | <1 | <0,03 | <0,5/9,5 |
| RdKW20 WT | 0,25 | <0,5/0,25 | 2 | <0,25 | <0,06 | <0,12 | 0,5 | <0,25 | <1 | <1 | <0,03 | >2/38 |
| RdKW20Δ*vacJ* | 0,25 | <0,5/0,25 | 1 | <0,25 | <0,06 | <0,12 | 0,5 | <0,25 | <1 | <1 | <0,03 | >2/38 |

**Table S2.** *H. influenzae* minimal medium free of fatty acids (MM-FFA): composition and preparation.

<table>
<tr>
<td rowspan="2">S21 (500 ml):<br>L-Aspartic Acid 2 g<br>L-Glutamic Acid 0,1 g<br>Fumaric Acid 0,5 g<br>NaCl 2,35 g<br>$K_2HPO_4$ 0,435 g<br>$KH_2PO_4$. 0,335 g<br>Tween80 0,1 ml<br>Ajust pH to 7.4 with NaOH 4N.<br>Add $dH_2O$ to 500 ml.<br>Autoclave and store at RT.</td>
<td rowspan="2">S22 (100 ml):<br>L-Cystine 0,04 g<br>L-Tyrosine 0,1 g<br>Dissolve in 10 ml of HCl 1N at 37ºC.<br>Add $dH_2O$ M-Q to 100 ml.<br>Add to this mixture:<br>L-Citruline 0,06 g<br>L-Phenylalanine 0,2 g<br>L-Serine 0,3 g<br>L-Alanine 0,2 g<br>Sterilize by filtration.<br>Store at 4ºC.</td>
<td>S23 (100 ml):<br>$CaCl_2$ 1,1099 g<br>$dH_20$ 100 ml<br>Autoclave.<br>Store at 4ºC.</td>
</tr>
<tr>
<td>S24 (100 ml):<br>$MgSO_4$ 2,4648 g<br>$dH_20$ 100 ml<br>Autoclave.<br>Store at 4ºC.</td>
</tr>
<tr>
<td colspan="3">MM-FFA preparation:<br>1) Mix S21:S22:S23:S24 (volume rate, 100:1:1:1)<br>2) Supplement the mix with hemin (10 μg/ml) and β-NAD (10 μg/ml)<br>3) If necessary, add glucose 20 mM (stock solution: glucose 1M prepared in distilled water and sterilized by filtration)</td>
</tr>
</table>

**Table S3.** Distribution and level of conservation of genes involved in fatty acid uptake, degradation and recycling in *H. influenzae* (protein BLAST).

| | *E. coli* gene | Gene ID | Protein ID | Hi gene | Gene ID RdKW20 | Protein ID RdKW20 | % identity *E. coli* | Gene ID NTHi375 | Protein ID NTHi375 | % identity *E. coli* |
|---|---|---|---|---|---|---|---|---|---|---|
| **Fatty acid degradation** | *fadL* | b2344 | NP_416846.2 | *fadL* | HI0401 | NP_438563.1 | 40% | NF38_03050 | AIT67229.1 | 39% |
| | *fadD* | b1805 | NP_416319.1 | *fadD* | HI0390.1 | NP_438551.1 | 65% | NF38_03105 | AIT67240.1 | 65% |
| | *fadE* | b0221 | NP_414756.2 | --- | --- | --- | --- | --- | --- | --- |
| | *fadB* | b3846 | NP_418288.1 | --- | --- | --- | --- | --- | --- | --- |
| | *fadA* | b3845 | YP_026272.1 | --- | --- | --- | --- | --- | --- | --- |
| **Membrane lipid asymmetry maintenance** | *mlaA* | b2346 | NP_416848.1 | *vacJ* | HI0718 | NP_438876.1 | 42% | NF38_01540 | AIT66950.1 | 42% |
| | *mlaB* | b3191 | NP_417658.4 | *mlaB* | HI1083 | NP_439240 | 32% | NF38_08695 | AIT68260 | 32% |
| | *mlaC* | b3192 | NP_417659.1 | *mlaC* | HI1084 | NP_439241 | 42% | NF38_08690 | AIT68259 | 43% |
| | *mlaD* | b3193 | NP_417660.1 | *mlaD* | HI1085 | NP_439242 | 60% | NF38_08685 | AIT68258 | 58% |
| | *mlaE* | b3194 | NP_417661.1 | *mlaE* | HI1086 | NP_439243 | 73% | NF38_08680 | AIT68257 | 72% |
| | *mlaF* | b3195 | NP_417662.1 | *mlaF* | HI1087 | NP_439244 | 67% | NF38_08675 | AIT68256 | 67% |
| | *pqiA* | b0950 | NP_415470.1 | *pqiA* | HI1671 | NP_439813.1 | 37% | NF38_05885 | AIT67735.1 | 37% |
| | *pqiB* | b0951 | NP_415471.1 | *pqiB* | HI1672 | NP_439814.1 | 43% | NF38_05880 | AIT67734.1 | 43% |
| **Outer membrane phospholipase** | *pldA* | b3821 | NP_418265.1 | --- | --- | --- | --- | --- | --- | --- |
| **Acyl-transferases** | *lpxA* | b0181 | NP_414723.1 | *lpxA* | HI1061 | NP_439219.1 | 68% | NF38_08800 | AIT68281.1 | 68% |
| | *lpxD* | b0179 | NP_414721.1 | *lpxD* | HI0915 | NP_439075.1 | 65% | NF38_00400 | AIT66742.1 | 65% |
| | *lpxL* | b1054 | NP_415572.1 | *htrB* | HI1527 | NP_439676.2 | 55% | NF38_07450 | AIT68026.1 | 57% |
| | *msbB* | b1855 | NP_416369.1 | *msbB* | HI0199 | NP_438368.1 | 45% | NF38_04430 | AIT67476.1 | 45% |
| | *plsB* | b4041 | NP_418465.4 | *plsB* | HI0748 | NP_438907.1 | 57% | NF38_ 01210 | AIT66899.1 | 57% |
| | *plsC* | b3018 | NP_417490.1 | *plsC* | HI0734 | NP_438893.1 | 62% | NF38_ 01275 | AIT66912.1 | 62% |
| | *pagP* | b0622 | NP_415155.1 | --- | --- | --- | --- | --- | --- | --- |
